# Nannochloropsis, a rich source of diacylglycerol acyltransferases for engineering of triacylglycerol content in different hosts

**DOI:** 10.1186/s13068-016-0686-8

**Published:** 2017-01-03

**Authors:** Krzysztof Zienkiewicz, Agnieszka Zienkiewicz, Eric Poliner, Zhi-Yan Du, Katharina Vollheyde, Cornelia Herrfurth, Sofia Marmon, Eva M. Farré, Ivo Feussner, Christoph Benning

**Affiliations:** 1 Michigan State University-US Department of Energy Plant Research Laboratory, Michigan State University, East Lansing, MI 48824 USA; 2 Department of Plant Biochemistry, Albrecht-von-Haller-Institute for Plant Sciences, Georg-August-University, 37073 Göttingen, Germany; 3 Great Lakes Bioenergy Center, Michigan State University, East Lansing, MI 48824 USA; 4 Cell and Molecular Biology Program, Michigan State University, East Lansing, MI 48824 USA; 5 Dept. of Plant Breeding, Swedish University of Agricultural Sciences, Alnarp, Sweden; 6 Department of Plant Biology, Michigan State University, East Lansing, MI 48824 USA; 7 Department of Plant Biochemistry, Göttingen Center for Molecular Biosciences (GZMB), Georg-August-University, 37073 Göttingen, Germany; 8 Department of Plant Biochemistry, International Center for Advanced Studies of Energy Conversion (ICASEC), Georg-August-University, 37073 Göttingen, Germany; 9 Department Biochemistry and Molecular Biology, Michigan State University, East Lansing, MI 48824 USA

**Keywords:** *Nannochloropsis oceanica*, Microalgae, DGAT, Triacylglycerol, Lipid storage, Lipid droplets

## Abstract

**Background:**

Photosynthetic microalgae are considered a viable and sustainable resource for biofuel feedstocks, because they can produce higher biomass per land area than plants and can be grown on non-arable land. Among many microalgae considered for biofuel production, *Nannochloropsis oceanica* (CCMP1779) is particularly promising, because following nutrient deprivation it produces very high amounts of triacylglycerols (TAG). The committed step in TAG synthesis is catalyzed by acyl-CoA:diacylglycerol acyltransferase (DGAT). Remarkably, a total of 13 putative DGAT-encoding genes have been previously identified in CCMP1779 but most have not yet been studied in detail.

**Results:**

Based on their expression profile, six out of 12 type-2 DGAT-encoding genes (*NoDGTT1*-*NoDGTT6*) were chosen for their possible role in TAG biosynthesis and the respective cDNAs were expressed in a TAG synthesis-deficient mutant of yeast. Yeast expressing *NoDGTT5* accumulated TAG to the highest level. Over-expression of *NoDGTT5* in CCMP1779 grown in N-replete medium resulted in levels of TAG normally observed only after N deprivation. Reduced growth rates accompanied *NoDGTT5* over-expression in CCMP1779. Constitutive expression of *NoDGTT5* in *Arabidopsis thaliana* was accompanied by increased TAG content in seeds and leaves. A broad substrate specificity for NoDGTT5 was revealed, with preference for unsaturated acyl groups. Furthermore, NoDGTT5 was able to successfully rescue the Arabidopsis *tag1*-*1* mutant by restoring the TAG content in seeds.

**Conclusions:**

Taken together, our results identified *NoDGTT5* as the most promising gene for the engineering of TAG synthesis in multiple hosts among the 13 DGAT-encoding genes of *N. oceanica* CCMP1779. Consequently, this study demonstrates the potential of NoDGTT5 as a tool for enhancing the energy density in biomass by increasing TAG content in transgenic crops used for biofuel production.

**Electronic supplementary material:**

The online version of this article (doi:10.1186/s13068-016-0686-8) contains supplementary material, which is available to authorized users.

## Background

Microalgae have experienced a renaissance as a potential resource for biofuel production because many of them have the ability to store as much as 20–50% of their dry weight as lipids. In addition, they are highly efficient photosynthesizers and their culturing methods generally do not compete with agricultural food production. Algal storage lipids, i.e., TAGs, are produced under unfavorable environmental conditions when cells experience stress [1]. In plant and microalgae, synthesis of the fatty acids (FAs) takes place in the plastid, and synthesis of TAG occurs either at the endoplasmic reticulum (ER) or, as has recently been recognized, the plastid envelopes as well [2]. De novo synthesized TAGs are deposited in discrete spherical structures called lipid droplets (LDs) located in the cytoplasm or in case of plastoglobuli in the plastid [3].

The final step of TAG formation includes esterification of diacylglycerol (DAG) with acyl-CoA by acyl-CoA:diacylglycerol acyltransferase (DGAT) [4]. In eukaryotic organisms, two different types of membrane-bound DGAT enzymes are directly involved in TAG formation—type 1 (DGAT) and type 2 (or DGTT). Both have similar function, but their genes evolved separately [5]. In addition to the DGATs, phospholipid:diacylglycerol acyltransferase (PDAT) can convert DAG into TAG using phosphatidylcholine (PC) as an acyl donor. The PDAT-mediated pathway of TAG formation has been characterized in yeast and plants [6, 7]. Most of the current knowledge of TAG synthesis, accumulation, and cellular trafficking in microalgae is derived from the analysis of *Chlamydomonas reinhardtii*, which has been extensively studied at the molecular and biochemical level [8, 9]. However, *Chlamydomonas* is generally not considered to be an oleaginous alga. Moreover, recent molecular studies suggest that some aspects of lipid metabolism may differ between *Chlamydomonas* and oleaginous microalgae, reflecting the broad evolutionary diversity of microalgae [1, 2]. Therefore, other microalgae that have a greater production capacity for TAG have been directly targeted for gene functional analysis and genetic engineering of lipid metabolic pathways. One of the emerging models is *Nannochloropsis*, which is a prodigious producer of both, lipids and biomass. *Nannochloropsis* species are small unicellular heterokont algae living in marine, fresh, or brackish water. Their lipid content is especially high following nitrogen (N) deprivation [10–13].

Key genes governing TAG synthesis in algal cells have been identified among the annotated genomes by comparing global gene expression between N-replete and N-deprived cells [14, 15]. In the *Chlamydomonas* genome, five putative DGAT-encoding genes are present but only one of them (*DGTT1*) was found to be up-regulated under N deprivation [14, 15]. Functional characteristics of *Chlamydomonas* DGTTs revealed their broad substrate specificity and ability to increase the TAG content in vegetative tissues of higher plants [16, 17]. The genome of the unicellular photoautotrophic green alga *Ostreococcus tauri* encodes three putative type 2 DGAT-like proteins but none with similarity to type 1 DGAT [18]. Nannochloropsis is a remarkable exception among currently studied microalgae since 13 putative DGAT-encoding genes were identified in the genomes of two *N. oceanica* strains, CCMP1779 [19] and IMET1 [20]. In the first strain, we previously identified only one gene possibly encoding a protein similar to the plant type 1 DGAT, while putative type 2 DGATs are likely encoded by 12 genes. Thus far, a biological rationale for such a large DGTT gene family in *N. oceanica* is not clear. However, we hypothesize that the expansion of this specific gene family contributes to the extraordinary capability of this organism to accumulate oil to high amounts and perhaps advanced mechanism to regulate this process [19]. As reported recently, the over-expression of one of the type 2 DGAT-encoding cDNAs of *Nannochloropsis oceanica* resulted in elevated levels of TAG in this microalgae [21].

Here, we assess the predicted DGAT-encoding gene family of *N. oceanica* CCMP1779 and focus on the gene showing the highest gene expression, *NoDGTT5*. Expression of this gene not only restores the lipid phenotype in TAG-deficient mutants of yeast and *Arabidopsis* but also causes over-production of TAG in *N. oceanica* CCMP1779, tobacco leaves as well as in non-seed and seed tissue of wild-type Arabidopsis.

## Results

### Transcriptional profiling of *N. oceanica* CCMP1779 DGAT genes

**Fig. 1:**
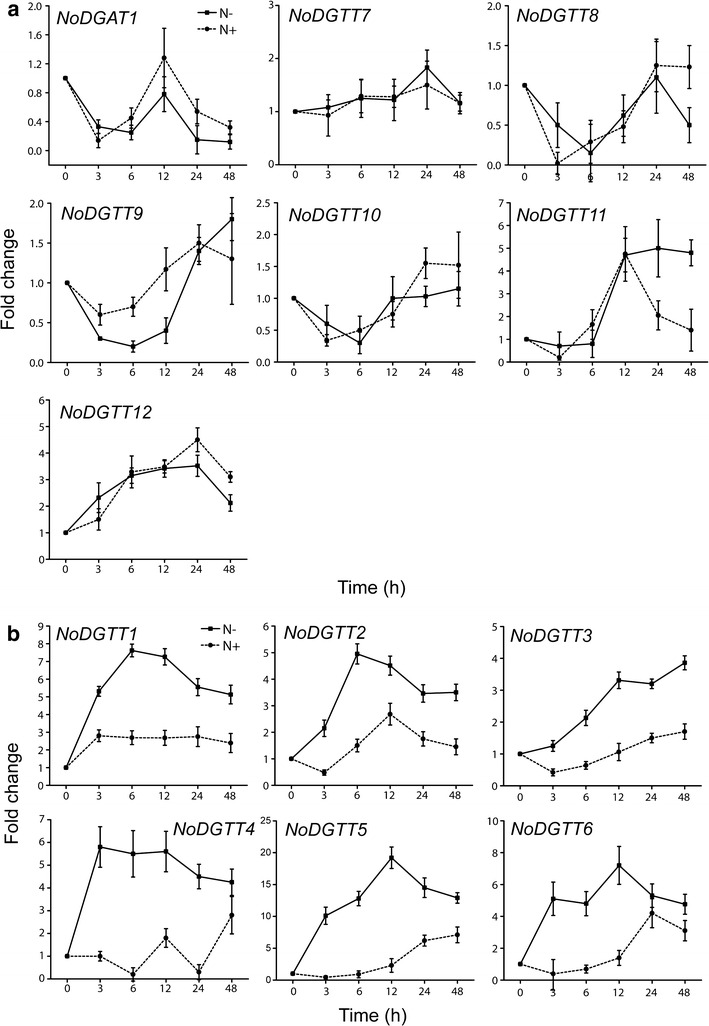
Transcript dynamics of the predicted *DGAT* genes in *N. oceanica* growing in nitrogen-replete (N+, *dashed line*) and nitrogen-depleted (N−, *solid line*) medium for 48 h analyzed by qPCR. Relative transcript levels are expressed as a fold change during 48 h after transfer to N-deprived conditions with respect to time 0. No significant differences in expression was found for *DGAT1* and *DGTT7*–*DGTT12* genes in response to N deprivation (**a**). The genes *DGTT1* through *DGTT6*, which are up-regulated following N deprivation, (**b**) were chosen for further functional analysis. Each data point represents the average of three biological replicates. Each sample was analyzed in technical triplicates. Values represent mean ± SD (*n* = 3)

The genome of *N. oceanica* CCMP1779 encodes one putative type 1 DGAT (*NoDGAT1*) and 12 putative type 2 DGAT genes (here referred as *NoDGTT1*–*NoDGTT12*) [19]. To compare the expression in response to N deprivation when TAG biosynthesis is highest [19], the transcriptional profile of the 13 DGAT genes was analyzed by qPCR under N-replete and N-deprived conditions under constant light using the actin gene as reference gene [22] (Fig. 1). Based on the observed expression patterns, the 13 analyzed DGAT genes were categorized into two groups. The first includes *NoDGAT1* and six type 2 DGAT-encoding genes (*NoDGTT7*–*NoDGTT12*) (Fig. 1a), which were unaffected by N deprivation. The second group consists of the remaining six type 2 DGAT-encoding genes (*NoDGTT1*–*NoDGTT6*) with increased transcript levels following N deprivation (Fig. 1b). Differences between the two conditions were greatest for *NoDGTT4* already 3 h after N-deprivation, whereas *NoDGTT1* and *NoDGTT2* reached their highest levels of expression after 6 h. During extended N deprivation, the abundance of most transcripts gradually decreased, with the exception of the expression of *NoDGTT3*, which increased with culture time and reached the highest level after 48 h. In response to N deprivation, transcript levels of *NoDGTT5* and *NoDGTT6* were transiently increased, with a maximum at 12 h. The highest fold change in expression among all the analyzed DGAT genes was observed for *NoDGTT5*, with a 20-fold increase at its maximum.

### Sequence analysis of *N. oceanica* CCMP1779 DGTTs

As a first step to predict their function, conserved amino acid residues and motifs were identified by multiple sequence alignments of *N. oceanica* CCMP1779 DGATs [19]. Sequences of NoDGAT1 as well as of type 1 DGATs from Arabidopsis, human, mouse, and rat were compared. Within the alignment conserved amino acid residues are located in the C-terminus of all the proteins (Additional file 1: Figure S1). Additional file 2: Figure S2 shows the seven conserved sequence motifs found in type 1 DGATs: GL, KSR, PTR, QP, LWLFFEFDRFYWWNWWNPPFSHP, FQL, NGQPY. Only two of the seven sequence motifs are fully conserved in NoDGAT1.

The type 2 DGAT proteins from *N. oceanica* were aligned with orthologues from Arabidopsis and human. Similar to type 1 DGATs, all conserved regions in the type 2 DGATs are located in the C-terminus of the proteins (Additional file 3: Figure S3). All six conserved sequence motifs of type 2 DGATs from other organisms were present in *N. oceanica* DGTTs as shown in Additional file 4: Figure S4. Those include: PH, PR, GGE, RGFA, VPFG, and G blocks. Seven out of the 16 highly conserved amino acids present in all the other type 2 DGATs are conserved in DGTTs from *N. oceanica* (Additional file 4: Figure S4). These include the proline and phenylalanine residues. Of those motifs, only the PH block is present in all NoDGTTs without any amino acid changes (Additional file 4: Figure S4, red asterisks).

Besides the six major motifs present in type 2 DGATs, two minor motifs—YFP and the flanking region of the PH block, are thought to contain residues essential for DGAT activity in yeast [23]. No perfectly conserved YFP motif has been found in *N. oceanica* CCMP1779 type 2 DGATs (Additional file 5: Figure S5). The region flanking the PH block provides certain hints about phylogenetic relations between type 2 DGATs. In plants, this region is composed of the E-PH-S motif, whereas in animals the sequence H-PH-G is usually present. In *N. oceanica* CCMP1779, the animal-like H-PH-G sequence is more common (Additional file 5: Figure S5). NoDGTT5, NoDGTT7, and NoDGTT10 show different hydrophobicity patterns within the PH block flanking region due to the presence of a hydrophobic amino acid instead of a polar at the first position.

### Expression of *NoDGTTs* in a TAG-deficient yeast

**Fig. 2:**
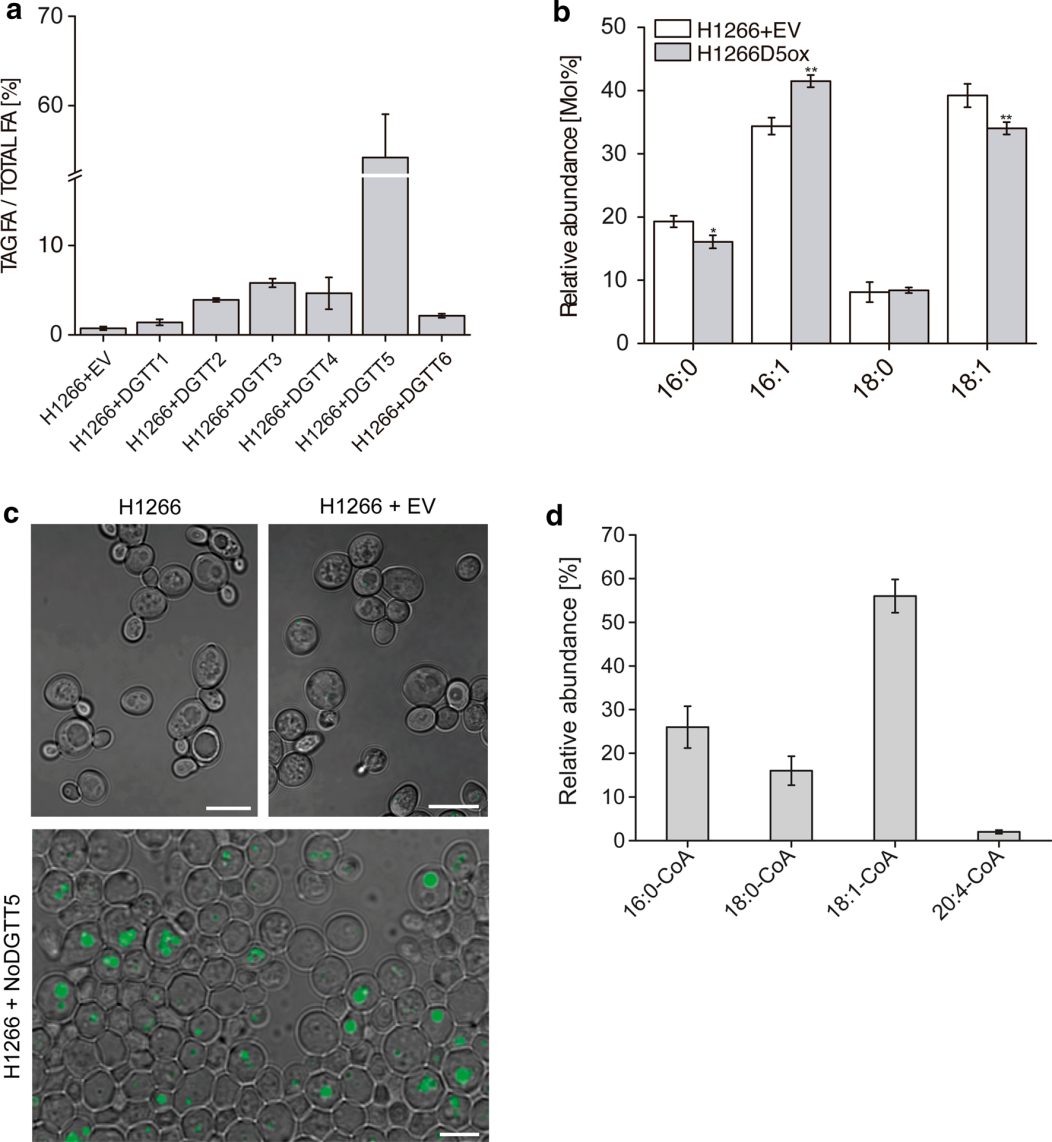
Restoring of TAG synthesis in H1266 yeast mutant by *N. oceanica DGTT1*-*6* and *PDAT1* over-expression. **a** Quantification of TAG levels extracted from transformed yeast shown as fatty acids esterified to TAG (TAG FA) over total fatty acids (FA total). DGTT proteins were present in each case but varied as determined by immunoblotting (Additional file 7: Figure S6). Tripentadecanoin (tri15:0) TAG was added as internal standard (*n* = 3; average ± SD). H1266 + EV indicates the empty vector control. **b** Changes in the fatty acid profile between wild-type and transformed yeast. **c** Lipid droplets formation analyzed by Nile Red staining (*green*) in H1266 cells, H1266 strain transformed with empty vector and H1266 cells expressing *NoDGTT5* gene. *Bars* 5 μm. **d** Competition assay with unlabeled substrate CoAs. The composition of synthesized TAG was analyzed by UHPLC-nano ESI-MS/MS and is given as relative content of each used CoA in total pool of newly synthesized TAG. For all experiments values represent mean ± SD (*n* = 3)

Because of their high expression following N-deprivation, six type 2 DGAT-encoding genes (*NoDGTT1* through *NoDGTT6*) were chosen for further functional studies. To test their activity, we expressed the full-length coding sequences of *NoDGTT1*–*NoDGTT6* in the TAG-deficient yeast mutant H1266 (Fig. 2). The full coding sequences from this study were deposited in GenBank (Accession numbers given in Additional file 6: Table S2). The yeast H1266 mutant strain contains null alleles for three of the four genes encoding enzymes with DGAT activity (*DGA1*-diacylglycerol acyltransferase 1, *LRO1*-lecithin: cholesterol acyl transferase 1, and *ARE2*-acyl-coenzyme A:cholesterol acyl transferase 2) [24], resulting in cells with only 1% of their wild-type DGAT activity.

We confirmed the presence of the NoDGTT1-6 proteins in H1266 cells by immunoblotting (Additional file 7: Figure S6). The amount of TAG produced was estimated by thin-layer chromatography (TLC) and gas chromatography-mass spectrometry (GC–MS) performed on lipid extracts from the transgenic yeast (Fig. 2a). Yeast producing NoDGTT2, NoDGTT3, and NoDGTT4 showed much higher TAG levels (4.2, 4.8, and 5.2% of total lipids, respectively) when compared to the empty vector control (0.61% of total FA content) (Fig. 2a). NoDGTT1 and NoDGTT6 minimally increased TAG accumulation when compared to other analyzed DGTTs (1.6 and 2.6% of total FA content, respectively). Expression of *NoDGTT5* resulted in the most efficient restoration of the TAG production reaching 53% of total FAs. Additional lipid analysis showed that NoDGTT5 production in H1266 is accompanied by an increase in the proportion of 16:1 fatty acids in TAG, while 16:0 and 18:1 in TAG decreased (Fig. 2b). The content of 18:0 was similar between empty vector control and *NoDGTT5*-expressing transformants.

TAG levels in the *NoDGTT5*-expressing transformants were positively correlated with the number and size of lipid droplets formed by yeast cells. BODIPY staining (Fig. 2c; Additional file 7: Figure S6) with confocal scanning laser microscopy (CLSM) and ultrastructural analysis of transgenic yeast by transmission electron microscopy (TEM) (Additional file 7: Figure S6) confirmed the accumulation of TAG in lipid droplets. H1266 cells and H1266 yeast transformed with the empty vector had no LDs, but showed a central vacuole as the most prominent cellular organelle (Fig. 2c; Additional file 7: Figure S6). The most numerous and prominent LDs were observed in the NoDGTT5-producing yeast. H1266 cells expressing *NoDGTT1*–*NoDGTT4* had less numerous and smaller LDs than those expressing *NoDGTT5*.

### In vitro activity of NoDGTT5

The activity of NoDGTT5 was determined in two in vitro assays using microsomes isolated from yeast expressing NoDGTT5 or containing an empty control vector. In the first assay, a mixture of equal amounts of 16:0-CoA, 18:0-CoA, 18:1-CoA, and 20:4-CoA together with di-6:0-DAG was fed to the microsomes extracted from H1266 expressing NoDGTT5 and the composition of formed TAGs harboring two 6:0 residues and one additional fatty acid were analyzed by UPLC-MS/MS. The NoDGTT5 containing microsomes had the highest proportion of TAG containing 18:1-CoA over the 18:0-CoA and 16:0-CoA and the lowest levels in case of 20:4 (Fig. 2d). No TAG was detected in the assay using microsomes from H1266 cells containing the empty control vector (data not shown). In the second assay, microsomes were incubated with equimolar amounts of ^14^C-labeled palmitoyl-CoA (16:0-CoA), stearoyl-CoA (18:0-CoA), oleoyl-CoA (18:1-CoA), α-linoleoyl-CoA (18:3-CoA), and arachidonoyl-CoA (20:4-CoA) along with di-6:0 DAG. The level of DGAT activity was estimated by measuring the levels of radioactivity of the resulting TAG band separated by TLC. As radiolabeled FFAs for 16:0-CoA and 18:0-CoA were present after TLC separation of both, substrates alone (data not shown) and lipids separated from the reaction mixture, we excluded the possibility of breakdown of these CoAs to FFAs during enzymatic assay and eventual loss of substrate content. Among the five used CoAs, the strongest incorporation was observed for 18:3-CoA and 18:1-CoA, respectively (Additional file 8: Figures S7A and S7B). In case of microsomes fed with 16:0-CoA and 18:0 CoAs, the intensity of the TAG band was significantly lower, meanwhile the incorporation was lowest for 20:4-CoA. For all substrate CoAs, no radiolabeled TAG band was detected for microsomes extracted from H1266 containing the empty control vector. Together, our data suggest that NoDGTT5 is able to incorporate varying acyl-CoA species into TAG with a stronger preference towards unsaturated acyl groups with a chain length of 18 carbon atoms.

Additionally, to test if the substrate availability might be a determining factor for the product specificity of NoDGTT5, we analyzed the relative content of acyl-CoA species in non-transgenic lines of *N. oceanica* CCMP1779 as well as in leaves of tobacco and *Arabidopsis*. The obtained data were compared with the substrate/product profiles observed in the NoDGTT5-expressing lines of these hosts (Additional file 8: Figure S7C). We observed that in *N. oceanica* CCMP1779 the main acyl-CoA species were 16:1/18:3/20:5, 16:0 and 18:2/20:4, respectively. In turn, saturated acyl-CoAs were mostly present in the Arabidopsis leaf, with 16:0, 20:0, 24:0 and 26:0 as the main species. Similarly, 16:0-CoA was also the most abundant in tobacco leaves; however, a relatively high content of unsaturated 16:1/18:3/20:5 acyl-CoAs as well as of 26:0-CoA were also found (Additional file 8: Figure S7C).

### Over-expression of *NoDGTT5* in *N. oceanica* CCMP1779

As *NoDGTT5* was the most efficient in restoration of TAG synthesis in H1266, we over-expressed it in *N. oceanica* CCMP1779 cells. To investigate the efficiency of NoDGTT5 in TAG synthesis and its subcellular localization in *N. oceanica* CCMP1779 under N+ and N− conditions, we over-expressed the full coding sequence of *NoDGTT5* (GenBank Accession number KY273672) using two types of constructs. In the first, we employed the strong, nearly constitutive elongation factor (EF) promoter (Additional file 9: Figure S8A). A Venus fluorescent protein (VFP) was fused to the protein as a reporter tag. In the second vector (Additional file 9: Figure S8B), the EF promoter was replaced by the native promoter sequence of *NoDGTT5* and green fluorescent protein (GFP) was fused to the protein as a reporter.

**Fig. 3:**
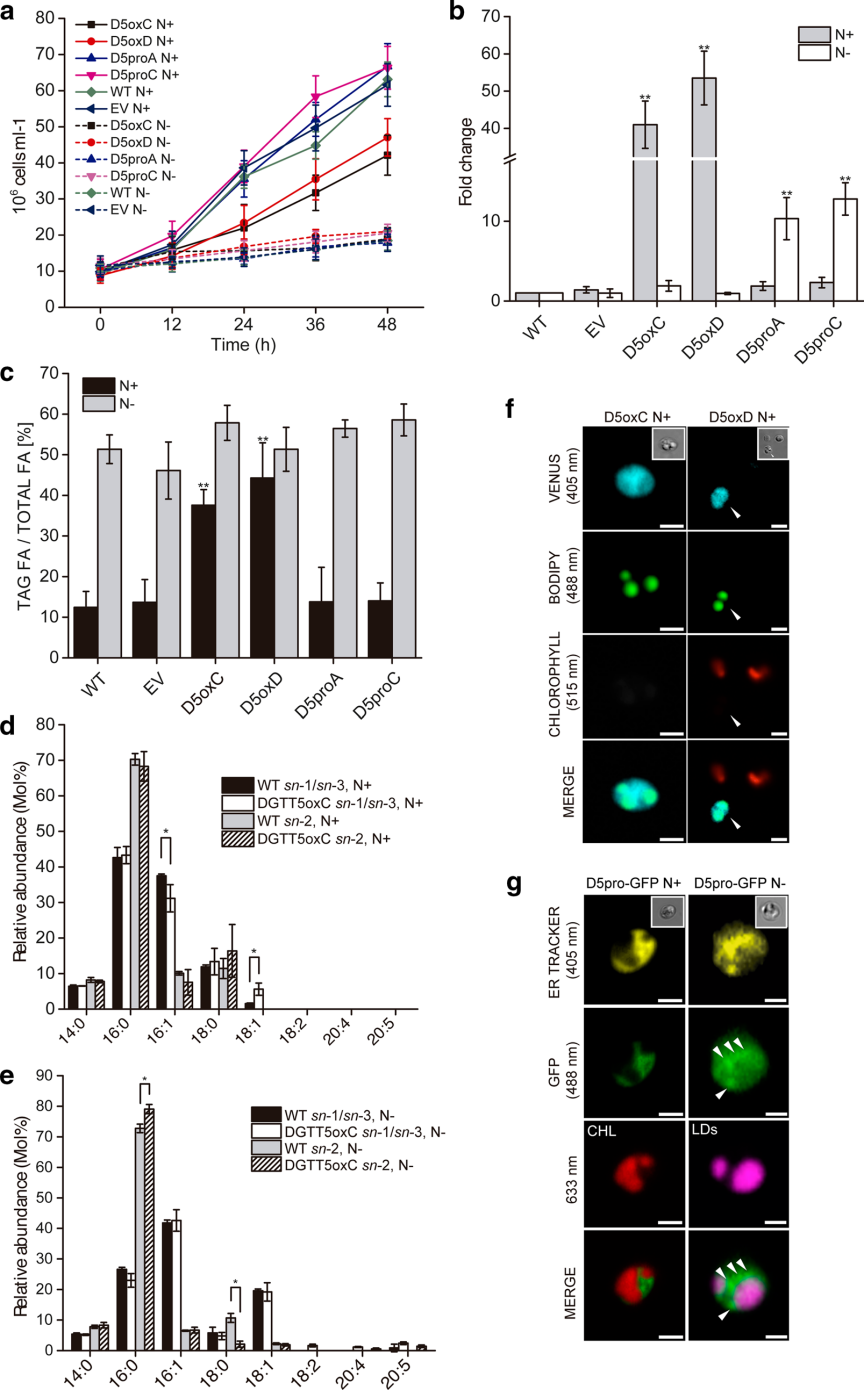
Over-expression and expression of *NoDGTT5* in *N. oceanica* CCMP1779 under control of the EF promoter (D5oxC and D5oxD) and the native promoter (D5proA and D5proC), respectively. **a** Growth of wild-type stain (WT), control vector transformants (EV), and lines over-expressing *NoDGTT5* under N-replete (N+) and N-depleted (N−) conditions. **b** Relative expression of *NoDGTT5* analyzed by qPCR in transformants growing under N+ and N− conditions. qPCR values were normalized to the *ACTIN* qPCR values and to the expression level of the wild-type under the respective condition (N+ or N−). Each data point represents the average of three biological replicates. Each sample was analyzed in technical triplicates. Values represent mean ± SD (*n* = 3) and asterisks indicate *P* < 0.01. **c** Relative abundance of TAG in strains used in **b**. **d**, **e** Positional analysis of TAG acyl groups of the WT strain and *NoDGTT5* over-expressing line growing in N+ (**d**) and N− (**e**) media. Data are presented as mean ± SD (*n* = 4–6), with *asterisks* indicating *P* < 0.05. **f** Detection of venus fluorescence in over-expressing lines D5oxC and D5oxD by using CLSM. *Bars* 1.5 μm. **g** Localization of NoDGTT5-GFP constructs expressed under native promoter in D5proA line; *CHL* chlorophyll; *LDs* lipid droplets. *Bars* 1.5 μm

We determined growth rates of transformed lines under both N+ and N− conditions (Fig. 3a). The cultures were pre-grown in liquid F/2 medium in order to obtain similar starting cell densities (20 × 10^6^ cells/ml) and afterwards half of the cultures were transferred into N-depleted medium and the other half continued growth in N-replete medium. Cell densities were measured every 12 h for 48 h. Under N+ conditions, the cultures containing cells over-expressing *NoDGTT5* under the control of the EF promoter showed nearly 50% lower growth rates than the wild-type and empty vector controls. Meanwhile, the cultures of transformants expressing *NoDGTT5* under its native promoter grew very similarly to the wild-type and empty vector controls. For all the lines, following N deprivation growth rates were similarly reduced and cell densities remained below 20 × 10^6^ per ml (Fig. 3a).

**Fig. 4:**
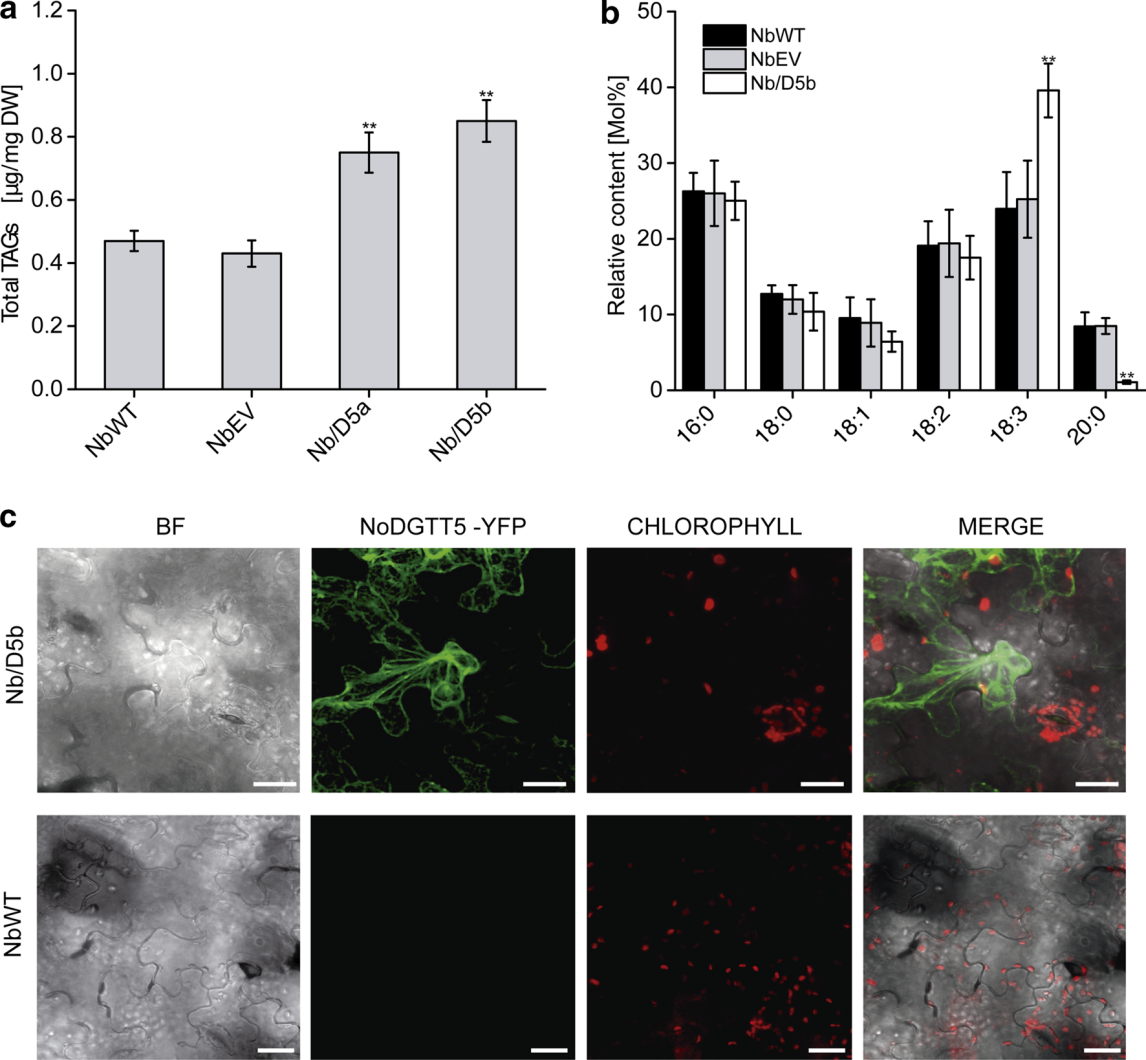
Transient expression of the *NoDGTT5* in tobacco leaves. **a** Total TAG content in non-transformed (NbWT), control (NbEV) and transformed leaves (Nb/D5a-Nb/D5b). **b** Composition and abundance of TAG species in non-transformed (WT), control (EV), and transformed leaf (Nb/D5a). For **a** and **b** averages of three replicates are shown with *error bars* indicating SD, with *asterisks* indicating *P* < 0.01. **c** Subcellular location of NoDGTT5-YFP (*green*) in a transiently transformed leaf (*top panel*). Lack of the fluorescence is observed in non-transformed leaves (*bottom panel*). *Bars* 25 μm

Of five transgenic lines over-expressing *NoDGTT5* under the EF-promoter, two with the highest levels of NoDGTT5-VFP (D5oxC and D5oxD, Additional file 10: Figure S9A, arrowheads) were chosen for further studies. Expression of *NoDGTT5* in *N. oceanica* CCMP1779 was shown to differ between lines expressing the transgene from the strong EF- and the weaker native promoters and was strongly dependent on culture conditions (Fig. 3b). When normalized to wild-type strain, a 50-fold increase in *NoDGTT5* transcript levels was observed in cultures with EF promoter-driven expression under N-replete conditions (line D5oxD), whereas only incremental or no increases in *NoDGTT5* expression occurred following N deprivation with respect to the wild-type and empty vector control. The opposite pattern of *NoDGTT5* transcript accumulation, albeit at ∼30% lower levels, was observed in cultures with cells expressing the transgene under the control of the native promoter (Fig. 4b, D5proA + D5proC). From 10- to 15-fold increase in *NoDGTT5* transcript abundance was detected under N-depleted conditions and only a slight increase of the expression was found under N-replete conditions, when normalized to wild-type strain.

EF-promoter-driven expression of *NoDGTT5* led to a strong increase of TAG content in lipid extracts under N-replete conditions, but not following N-deprivation (Fig. 3c). This effect was not observed in transgenic lines expressing *NoDGTT5* under control of its native promoter. To gain more information on the TAG synthesized by transgenic *N. oceanica* CCMP1779 over-expressing *NoDGTT5* under control of the EF promoter, we performed positional analysis of TAG acyl groups from cells cultured under both N+ and N− conditions. Hydrolysis of TAG by *Rhizopus arrhizus* lipase was used in this assay and revealed considerable changes in the composition of *sn*-2 and *sn*-1/*sn*-3 position of TAG between the transgenic line (D5oxC) and the wild-type strain. In both cases, the *sn*-1/*sn*-3 positions were occupied mostly by 16:0 and 16:1 acyl groups, whereas the *sn*-2 position of TAG mainly contained 16:0 acyl groups under N-replete conditions (Fig. 3d). In terms of the *sn*-1*/sn*-3 positions, a decrease in 16:1 and an increase in 18:1 was found in *NoDGTT5* over-expressing lines. The *sn*-2 position of TAG was mostly composed of 18:0 and no differences between distinct acyl group content were observed (Fig. 3d, e) under either N+ or N− conditions. No differences were observed in *sn*-1*/sn*-3 position between *NoDGTT5* over-expressing lines and wild-type strain under N-deprived conditions. However, the overall pattern of their contribution to the TAG pool changed, since 16:1 in *sn*-1/*sn*-3 positions was the most abundant, meanwhile a lower content of 16:0 and higher amount of 18:1 were found in both transgenic lines and the wild-type strain. The most prominent differences were observed for *sn*-2 composition patterns, since a higher content of 16:0 and a lower of 18:0 acyl groups was found in the *NoDGTT5* over-expressing lines.

Both lines over-expressing *NoDGTT5* under the control of the EF-promoter (D5oxC and D5oxD) were analyzed by using CLSM (Fig. 3f). We confirmed the presence of the NoDGTT5 protein fused to the Venus fluorescent protein (VFP) and formation of prominent LDs by transgenic *N. oceanica* CCMP1779, whereas no VFP-derived fluorescence was observed in the controls (Additional file 10: Figure S9B). Notably, some of the VFP-positive cells were found to have strongly reduced chloroplasts (Fig. 3f).

Microscopic analysis of *N. oceanica* CCMP1779 cells expressing *NoDGTT5* under control of its native promoter (Fig. 3g), combined with ER Tracker labeling revealed ER-specific localization of the NoDGTT5-GFP product under both N+ and N− conditions, in contrast to the control lines (Additional file 10: Figure S9C). Moreover, N deprivation was accompanied by accumulation of NoDGTT5-GFP-derived signal in close spatial proximity to forming LDs (Fig. 3g, arrowheads).

### Ectopic expression of *NoDGTT5* in seed and vegetative plant tissues

**Fig. 5:**
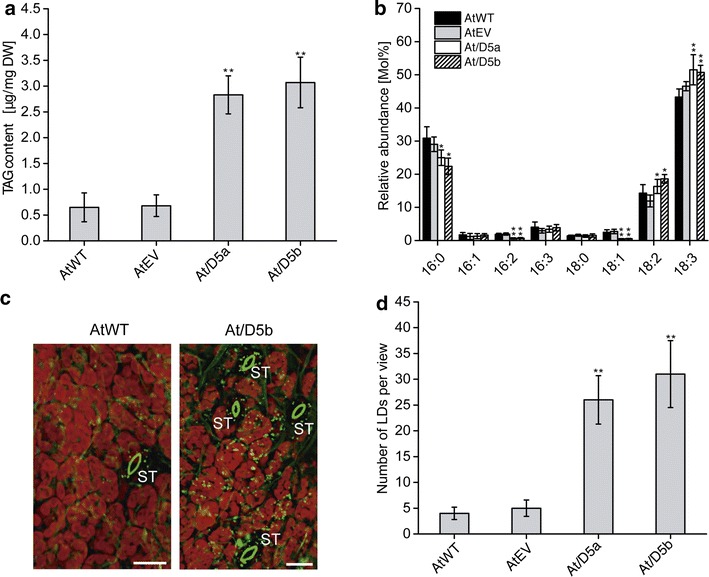
Ectopic expression of *NoDGTT5* in Arabidopsis leaves. **a** TAG content in wild-type (AtWT), control (AtEV), and *NoDGTT5* over-expressing lines (At/D5a and At/D5b). **b** Changes in TAG composition between wild-type, control, and *NoDGTT5* producing lines. **c** Representative confocal images of LDs labeled with BODIPY 493/503 (*green*) in the leaves of WT and transformed (At/D5b) Arabidopsis plants. *Red color* shows chlorophyll auto-fluorescence. Images are projections of Z-stacks of 28 optical sections taken 0.5 μm apart. *ST* stomata, *Bars* 25 μm. **d** LD counts in the leaves of WT, EV, and transformants expressing *NoDGTT5* after labeling with BODIPY 493/503. Values in **a**, **b** and **d** represent averages and SD of three individual experiments and *asterisks* indicate *P* < 0.01

To explore the utility of *NoDGTT5* for enhancing TAG synthesis in plant vegetative tissues, we expressed the full-length *NoDGTT5* coding sequence in tobacco leaves (Fig. 4) and in *Arabidopsis* (Fig. 5) under the control of the constitutive 35S cauliflower mosaic virus promoter, *35S:NoDGTT5*. Transient expression of *NoDGTT5* in tobacco leaves resulted in a doubling of FAs associated with TAG (Fig. 4a). Analysis of FA composition in TAG extracted from transformed leaves showed nearly a two-fold increase of 18:3 acyl chains and much lower 20:0 content (Fig. 4b). A C-terminal translational fusion of NoDGTT5 with YFP used for transient expression led us to approximate the subcellular distribution of NoDGTT5 in the epidermal cells of tobacco leaves. The recombinant NoDGTT5-YFP protein exhibited a fluorescence pattern consistent with endoplasmic reticulum location (Fig. 4c, upper row). Although taking into account that the relatively strong 35S promoter was used in this experiment, which can lead to over-expression artifacts, this result is nevertheless consistent with data observed for expression under the native promoter in *N. oceanica* CCMP1779 (Fig. 3g). No YFP fluorescence was found in the control (Fig. 4c, bottom row).

Seven over-expressing lines of Arabidopsis transformed with the *35S:NoDGTT5* construct (At/D5a–At/D5g) were carried forward to the T3 generation and used for estimation of *NoDGTT5* transcript level in the rosette leaves from 3-week-old plants (Additional file 11: Figure S10A). We used quantitative RT-PCR and analyzed the expression of *NoDGTT5* relative to *ACTIN2* as reference gene. The ratio of *NoDGTT5/ACTIN2* ranged from approximately 1 to almost 5 in the independent transgenic lines tested, while no transcripts were detected in the wild-type plants. Two lines with the highest *NoDGTT5* expression levels (At/D5a and At/D5b, Additional file 11: Figure S10A, arrowheads) were used for detailed analysis (Fig. 5). The TAG content in the leaves of At/D5a and At/D5b plants was more than threefold higher (2.7 and 3.2 μg/mg of DW, respectively) when compared to the wild-type plants and empty vector control (both 0.7 μg/mg of DW) (Fig. 5a). The increase in TAG levels correlated well with the relative abundance of *NoDGTT5* transcripts in both lines. Moreover, a decrease in 16:0 and 16:2 acyl group content and elevated levels of 18:2 and 18:3 acyl groups were found in both transgenic lines (Fig. 5b). To assess the effect of *NoDGTT5* on cellular organization of TAG accumulation in the leaves of the transgenic lines, we analyzed LD formation by confocal microscopy (Fig. 5c). As shown in the micrographs in Fig. 5c and quantified in Fig. 5d, *NoDGTT5* expression was accompanied by a fivefold increase in LD numbers in leaf cells.

**Fig. 6:**
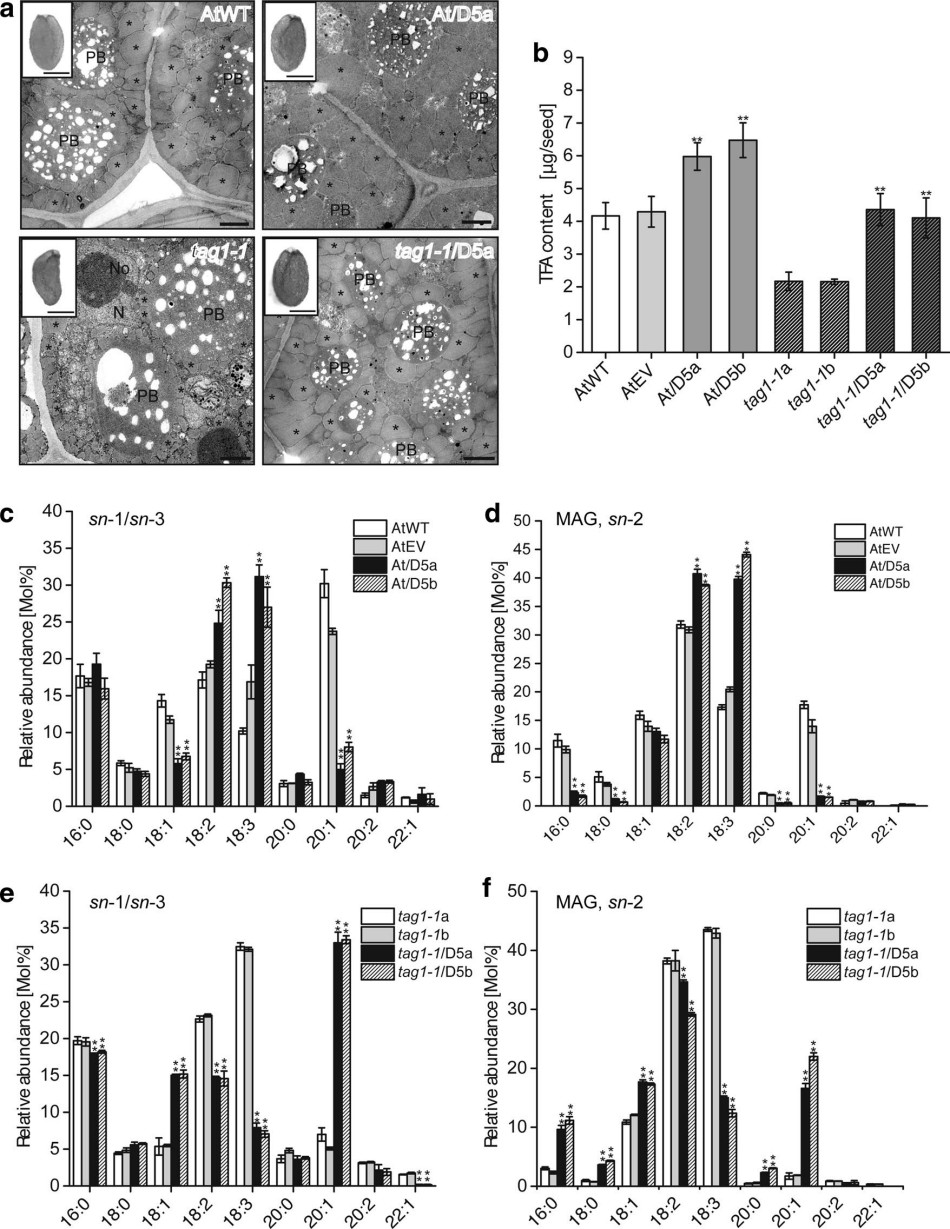
Constitutive expression of *NoDGTT5* in seeds of WT and *tag1*-*1 Arabidopsis* plants. **a** Morphology (inserts) and ultrastructure of seeds before (AtWT and *tag1*-*1*) and after (At/D5a and *tag1*-*1*/D5a) expressing of *NoDGTT5*. N; nucleus, No; nucleolus, PB; protein body, *asterisks* indicate lipid droplets. *Bars* in the inserts = 250 μm; *bars* in the micrographs = 1 μm. **b** TAG contents in the seeds from WT plants, plants transformed with control vector (AtEV), WT plants over-expressing *NoDGTT5* (At/D5a and At/D5b), mutant lines (*tag1*-*1*a and *tag1*-*1*b), and mutant lines complemented with *NoDGTT5* gene (*tag1*-*1*/D5a and *tag1*-*1/*D5b). **c**, **d** Positional analysis of TAG composition between WT plants, control plants (EV) and lines over-expressing *NoDGTT5* (At/D5a and At/D5b). **c** Content of *sn*-1/*sn*-3 moieties and **d** abundance of *sn*-2 moieties in respective TAG pools. **e**, **f** Positional analysis of TAG composition among mutant lines (*tag1*-*1*a and *tag1*-*1*b) and *tag1*-*1* lines complemented with *NoDGTT5* gene (*tag1*-*1*/D5a and *tag1*-*1*/D5b). In **b**–**f** averages of three replicates are shown with *error bars* indicating SD and with *asterisks* indicating *P* < 0.01

To test if *NoDGTT5* expression had any effect on the seed oil content, we analyzed the TAG levels and composition of seeds produced by the plants from *35S:NoDGTT5* lines At/D5a and At/D5b. We evaluated three different traits for seed yield in the line At/D5a: seed length, seed width, and the ratio of both (Table 1) and compared cellular organization of seed LDs between this line and the wild-type. *NoDGTT5*-expressing seeds were of slightly larger size. No substantial differences were found in LD number and size at the ultrastructural level of the seed cells (Fig. 6a, upper row); however, a slightly higher electron density of LDs was observed for D5oxA seeds. The transgenic seeds from all analyzed lines showed up to threefold higher expression of *NoDGTT5* than the wild-type plants (Additional file 11: Figure S10B). In the seeds of both of the over-expressing lines, the total fatty acid content per seed was higher than in the wild-type and empty vector control, reaching 6.09 and 6.33 μg, respectively (Fig. 6b). Moreover, positional analysis of TAG by using *R. arrhizus* lipase revealed several changes in the composition of FAs at the *sn*-2 and *sn*-1*/sn*-3 positions of the seed TAG between transgenic lines and the wild-type and empty vector controls (Fig. 6c, d). In the wild-type and empty vector control seeds, the *sn*-1/*sn*-3 position TAG contained high levels of 20:1 acyl groups, whereas the *sn*-2 positions were composed mostly of partially unsaturated C18 acyl groups. Over-expression of *NoDGTT5* resulted in a substantial increase of the 18:2 and 18:3 acyl groups and strong decrease in the 18:1 and 20:1 acyl groups in the *sn*-1/*sn*-3 pool of TAG (Fig. 6c). Similar changes were observed for the *sn*-2 positions of TAG, where decreases of 16:0, 18:0, and 20:0 acyl groups were found in the over-expression lines (Fig. 6d).

**Table 1:** Morphometry of the seeds from WT plants transformed with control vector (AtEV), WT expressing NoDGTT5 (At/D5a), mutant line (*tag1*-*1*), and mutant lines complemented with NoDGTT5 (*tag1*-*1*/D5a); number of measured seeds is given in parenthesis

Line	Length [μm]	Width [μm]	Length/width
AtEV	519 ± 31 (*248*)	319 ± 23 (*248*)	1.62 ± 0.39
At/D5a	539 ± 41 (*151*)	328 ± 18 (*151*)	1.62 ± 0.42
*tag1*-*1*	509 ± 22 (*210*)	266 ± 21 (*210*)	1.91 ± 0.12
*tag1*-*1*/D5a	512 ± 28 (*113*)	304 ± 31 (*113*)	1.68 ± 0.31

### Complementation of *Arabidopsis tag1*-*1* mutant with *NoDGTT5*

Since the *tag1*-*1* mutant has reduced DGAT activity resulting in less TAG content and an altered fatty acid composition [25, 26], we pursued the possibility of restoring its phenotype by ectopic expression of *35S:NoDGTT5*. Transformed lines with the highest levels of *NoDGTT5* expression in the seed (Additional file 11: Figure S10D) were chosen for further studies. Quantitative measurements of total FA content performed on mature seeds showed higher amounts of total FA in the *tag1*-*1 35S:NoDGTT5* complementation lines, when compared to *tag1*-*1* plants (Fig. 6b). In *tag1*-*1* seeds, the total FA content barely exceeded 2 μg per seed, whereas 35S-driven *NoDGTT5* expression increased total FA levels to 4 μg per seed, which is similar to the levels observed in wild-type seeds (Fig. 6b). Positional analysis of seed TAG by hydrolysis with *R. arrhizus* lipase was used to probe potential changes in TAG composition in response to *NoDGTT5* over-expression in *tag1*-*1* plants (Fig. 6e, f). For the *tag1*-*1* lines, we confirmed its characteristic FA composition of seed oil with high levels of 18:3 and greatly reduced amounts of 20:1 and 18:1 at *sn*-1/*sn*-3 and *sn*-2 positions of the TAG. A different pattern of FA composition was observed in the neutral lipid extracts from *tag1*-*1* plants expressing *NoDGTT5*. Great reduction of 18:3 as well as 18:2 levels was accompanied by a strong increase in the 20:1 content in both *sn*-1*/sn*-3 and *sn*-2 acyl groups, when compared to *tag1*-*1*. A higher content of 18:1 was also observed in *tag1*-*1* expressing *NoDGTT5*, in this case at the *sn*-1/*sn*-3 and *sn*-2 positions. In those lines, 16:0 and 18:0 were also more abundant in the *sn*-2 positions than in the *sn*-1/*sn*-3 (Fig. 6f).

Morphometric studies showed that the complementation of the seed TAG phenotype of *tag1*-*1* plants by expression of *NoDGTT5* was accompanied also by an increase in the seed size (Table 1). The *tag1*-*1* seeds expressing *NoDGTT5* reached the size observed for the wild-type plants. Ultrastructural analysis of the seeds revealed that *tag1*-*1* seeds possess strongly reduced LDs in size and number, whereas *tag1*-*1* expressing *NoDGTT5* formed seed LDs of size and density similar to the wild-type plants (Fig. 6a).

## Discussion

### Evolution of DGAT genes in *N. oceanica* CCMP1779

DGATs contribute to the bulk of TAG biosynthesis in eukaryotic cells by catalyzing the esterification of acyl-CoA to a diacylglycerol (DAG) moiety. DGAT has been proposed to be the rate-limiting enzyme of TAG accumulation. In Arabidopsis, there are two membrane-bound DGAT isozymes, encoded by *DGAT1* and *DGAT2* [27, 28]. Microalgae generally contain multiple copies of type 2 DGAT encoding genes (*DGTT*) and their number is remarkably diverse among algal genera. For example, three DGTT-encoding genes were found in *Ostreococcus tauri* [18], four in *Phaeodactylum tricornutum* [29, 30], five in *Chlamydomonas reinhardtii* [14, 15] and six in *Chlorella pyrenoidosa* [31]. *Nannochloropsis oceanica* sp. is one of the most remarkable examples of high copy numbers of *DGTT* genes [19, 20]. However, it should be noted that some of the predicted DGTTs could be in fact monoacylglycerol transferases (MGATs) or other type of acyltransferases, because it is difficult to distinguish them based only on sequence data.

We previously showed that predicted DGTT protein sequences of *N. oceanica* CCMP1779 are spread over the entire type 2 DGAT phylogenetic tree and do not form a distinct group [1]. They do not directly cluster with sequences from animals, plants or fungi, but with those from other algal groups and *Oomycetes*. Similar results were reported for other microalgal DGTTs [32]. Phylogenetic analyses revealed that in case of algal species with multiple DGTTs, they are more closely related to orthologues in other species than to their paralogues. A recent model of DGTT evolution in *N. oceanica* IMET1 assumed that out of the 11 DGTTs presumably encoded in its genome, six originated from a heterotrophic secondary host, four derived from a green algal ancestor, and one came from a red algae [20]. This model can also be adapted for the origin of *N. oceanica* CCMP1779 DGTTs, because some of the DGTTs appear to be related to their counterparts in green, and some in red algae and in *Oocmycetes* [1].

A diverse evolutionary origin of multiple DGAT-encoding genes in *N. oceanica* CCMP1779 could also explain their different expression patterns in response to N deprivation. Our results suggest that only NoDGTT1–NoDGTT6 might directly contribute to TAG accumulation in *N. oceanica* CCMP1779 cells following N depletion (Fig. 1b). Differential expression was also observed among *N. oceanica* IMET1 DGTT-encoding genes, where seven of eleven DGTT-encoding genes were up-regulated in response to N deprivation; however, considerable differences in their expression patterns were observed [22]. Up-regulation of selected DGTT-encoding genes following N deprivation was apparent also in other algal species [1], suggesting that not all of the encoded DGATs are equally involved in TAG formation in response to environmental stress, and some of them may be contributors to TAG synthesis under N+ conditions. The recent findings of Li et al. [21] seem to confirm this hypothesis, since the over-expression of type 2 DGAT-encoding cDNA corresponding to *NoDGTT7* (non-responsive to N starvation) in *N. oceanica* CCMP1779 resulted in an increased content of neutral lipids. Alternatively, as suggested previously [33] each of multiple DGAT-encoding genes might be activated by diverse effectors produced in response to the various TAG-inducing stress factors.

### Activity of NoDGTT5 in diverse hosts

NoDGTT5 contains all of the motifs found in the type 2 DGAT family [5, 32], with several single amino-acid variations. Four transmembrane domains were identified using bioinformatics tools, but no signal or targeting sequences were found, possibly because most of the programs are trained on land plant signal sequences. We addressed its ER-specific localization under both N+ and N− conditions in *N. oceanica* CCMP1779 cells (Fig. 3g) and in the epidermal cells of tobacco (Fig. 4c). Moreover, following N deprivation, NoDGTT5 accumulated in ER domains of *N. oceanica* CCMP1779 in close spatial proximity to forming LDs (Fig. 3g). Similar to plant and animal DGAT enzymes [34, 35], these results not only point to specific ER domains as the main location of NoDGTT5, but also as the main site of TAG synthesis directly involved in LD formation.

Expression of *NoDGTT5* in the H1266 yeast strain resulted in the most efficient restoration of TAG production and was accompanied by formation of the most prominent LDs when compared to other DGTTs from *Nannochloropsis* (Fig. 2). High efficiency in restoration of TAG synthesis in yeast TAG-deficient mutants was previously shown for other microalgal type 2 DGATs in *P. tricornutum* (*PtDGAT 2B*) [30], *C. reinhardtii* (CrDGTT2 and CrDGTT3) [16], and *O. tauri* [18]. Among *C. reinhardtii* DGTTs, the similar DGTT2 and DGTT3 isozymes were equally successful in restoring the TAG production in the H1266 strain [16]. Our sequence analysis showed only moderate similarity between the six DGTTs from *N. oceanica* CCMP1779 used for yeast expression (Additional file 4: Figure S4); thus, specific intrinsic features of NoDGTT5 that differentiate it from the other DGTTs could contribute to its efficiency in yeast cells. The YFH motif of DGTTs has been shown to be essential for the enzymatic DGAT activity in yeast [23] and is conserved in land plants and animals. However, none of the *N. oceanica* DGTTs possesses a fully conserved YFH motif (Additional file 5: Figure S5), which is consistent with reports for other microalgal species [32]. As shown in *O. tauri*, DGAT2A lacking the YFH motif was able to restore TAG biosynthesis in H1266, whereas the DGAT2C with a full YFH motif was unable to restore TAG production in yeast [18]. Collectively, these data suggest that the YFH motif may not be essential for DGAT activity as previously assumed. A motive that is more conserved in *N. oceanica* CCMP1779 DGTTs is the animal-like HPHG motif, as shown in Additional file 5: Figure S5. For this motif, NoDGTT5 is the only *N. oceanica* DGTT with a different first amino acid (M-PH-G). Similar variations were found previously for *Chlamydomonas*, and it was suggested that the first amino-acid residue of this motif may contribute to their specific catalytic activities [17], as was shown for yeast Dga1 and mouse DGAT2 enzymes [23, 36].

We were able to complement that the *Arabidopsis* mutant line *tag1*-*1*, which exhibits reduced DGAT activity, has reduced levels of 20:1 and 18:1, and accumulates 18:3 as the major fatty acid in TAG [25, 26]. The TAG content and composition in the *tag1*-*1* NoDGTT5 transformants were restored essentially to wild-type levels as confirmed by positional TAG analysis (Fig. 6). Previously, the *tag1*-*1* phenotype was successfully complemented by over-expression of endogenous *Arabidopsis* DGAT-encoding genes [37]. Similar to our findings, a positive correlation between the DGAT transgene expression and TAG content was reported. Complementation was also visible at organ- and ultrastructural levels. Seeds of *tag1*-*1* expressing *NoDGTT5* were of wild-type shape and size and had numerous large LDs. In the *tag1*-*1* lines expressing *NoDGTT5*, the TAG content was not as high as that observed in the wild-type expressing *NoDGTT5*. This may be due to the increase of DGAT activity in addition to the activity of endogenous DGATs in the transgenic wild-type seed, consistent with DGAT being rate-limiting for TAG biosynthesis in seeds.

### Effects of *NoDGTT5* expression on TAG composition

Only subtle changes in TAG composition were found in *NoDGTT5*-expressing *N. oceanica* CCMP1779, when compared to wild-type. In vitro lipase assays of extracts from cells grown in N-replete medium and over-expressing *NoDGTT5* showed a preference for 18:1 acyl groups at the *sn*-1/*sn*-3 positions, over more highly unsaturated acyl chains (Fig. 3d). Acyl-CoA profiling of *N. oceanica* CCMP1779 grown under N-replete conditions strongly suggested that substrate preference for NoDGTT5 does not depend on the availability of preferred substrates, since 18:1-CoA was underrepresented in the total pool of CoAs (Additional file 8: Figure S7C). Following N depletion, when the EF promoter activity is much lower, 16:0 acyl groups at the *sn*-2 position were preferred for TAG synthesis. This change in NoDGTT5 preference towards distinct substrates is an indirect evidence for the substrate flexibility of NoDGTT5, which allows it readily adjust to the actual metabolic state of the cell. The broad substrate specificity of NoDGTT5 was also confirmed by DGAT activity assays using yeast microsomes, for which both unsaturated and saturated acyl chains were used for TAG synthesis (Fig. 2d; Additional file 8: Figures S7A and S7B). As shown by expression profiling, a set of specific endogenous DGAT encoding genes is newly expressed following N deprivation in *N. oceanica* CCMP1779 and will be competing with NoDGTT5 for substrates to different extents, even though NoDGTT5 is likely the most abundant contributor based on its high transcript abundance. Comparison of TAG composition between wild-type and transgenic leaves suggested that mainly 18:3 FA as well as 18:2 acyl groups were used as substrates in tobacco and *Arabidopsis*, respectively. These observations stay in agreement with our results that NoDGTT5 is active towards a broad range of acyl-CoAs, but prefers those that are unsaturated (Fig. 2d; Additional file 8: Figures S7A, S7B). Such flexibility was observed for other DGAT enzymes, like *O. tauri* DGAT2B and yeast Dga1 but not for plant DGAT2 [18]. Introducing CrDGTT2 from *C. reinhardtii* into Arabidopsis also resulted in high levels of TAG accumulation in leaves and increased the relative abundance of 18:0 acyl chains in TAG [16]. Moreover, similar to *Nannochloropsis* cells, the NoDGTT5 preference in tobacco and *Arabidopsis* leaves does not seem to be dependent on the substrate availability as unsaturated acyl-CoAs were less abundant in both mentioned hosts (Additional file 8: Figure S7C). The most striking effect on TAG composition was seen in *Arabidopsis* seeds where in the wild-type 18:2 and 18:3 are increased and 20:1 decreased following NoDGTT5 expression, while the exact opposite is observed in the *tag1* background (Fig. 6c versus e and d versus f). Again, our data point to a strong preference of NoDGTT5 towards acyl-CoA substrates that are unsaturated, since the 18:2 and 18:3, at *sn*-2 position predominated in the stored TAGs. A similar, however slightly less obvious pattern, was observed for acyl-chains at the *sn*-1/*sn*-3 position. We interpret these changes in TAG composition to be the result of competition between seed endogenous DGATs and NoDGTT5. Collectively, it is likely that changes in TAG composition in the diverse *NoDGTT5*-expressing organisms also depends on the host contribution to the TAG pool. Experimental systems used in this study are of diverse evolutionary origin and reflect substantially different organization of lipid synthesis in terms of both quantity and composition. Thus, the observed apparent variations in NoDGTT5 substrate specificity between hosts likely also depend on the endogenous enzymatic equipment and individual organization of lipid accumulation pathways existing in the cell.

### Growth versus NoDGTT5-induced TAG accumulation

When we over-expressed *NoDGTT5* in *N. oceanica* CCMP1779 massive TAG synthesis was observed under N-replete conditions (Fig. 3). Similar results were also recently reported for other type 2 DGAT over-expressed in the cells of *N. oceanica* CCMP1779 [21]. Our results showed that lipid accumulation in *N. oceanica* CCMP1779 over-expressing *NoDGTT5* was accompanied by strongly reduced growth rates, appearance of LDs and chloroplast degradation (Fig. 3). Such cellular events normally occur in *N. oceanica* cells after transition from N+ to N− conditions [19]. These observations suggest that despite favorable environmental conditions, over-expression of *NoDGTT5* under the control of the EF promoter in *N. oceanica* CCMP1779 leads to cellular and metabolic changes normally observed during nutrient deprivation-induced quiescence. In this cellular state, cell divisions are temporarily halted and cell metabolism is reprogrammed to survival of the encountered adverse condition [38]. In microalgal cells, quiescence induced by N deprivation results in substantial disorganization of the photosynthetic apparatus, reduced rates of protein biosynthesis, up-regulation of lipase and autophagy genes, and accumulation of TAGs [38, 39]. Partial degradation of chloroplasts and cellular membranes likely accompany the redirection of cellular carbon partitioning mainly towards storage lipid synthesis as algal cells enter quiescence. The regulatory mechanisms or signals that allow algal cells to transition into and out of quiescence are poorly understood. Hence, it is striking that forcing *N. oceanica* CCMP1779 to accumulate high level of oils irrespective of nutrient availability resembles a cellular state with metabolic and structural changes encountered normally only during nutrient deprivation-induced quiescence. Whether this is simply a consequence of diverting carbon and resources from growth processes into TAG synthesis or whether metabolic signals as a result of increased TAG biosynthesis are causally involved remains an interesting question to be answered. Addressing this question is important to overcome the conundrum of the inverse relationship between biomass production and oil accumulation hampering the development of algal feedstocks.

## Conclusions

From a biotechnological viewpoint, we have demonstrated that *Nannochloropsis* DGAT-encoding genes are useful tools for manipulating TAG accumulation in microalgae, and seed and non-seed tissues in plants. Successful over-expression of *NoDGTT5* in *N. oceanica* CMP1779 cells resulted in a nearly threefold higher accumulation of TAGs during growth in N replete media (Fig. 3c). Overcoming the necessity of nutrient stress treatment for TAG accumulation is desirable for the robust production of microalgal industrial feedstocks. Optimization of DGAT expression systems may help balancing high TAG levels and growth rates for promising oleaginous microalgal strains, but ultimately, the underlying regulatory mechanism needs to be understood. We also demonstrated threefold accumulation of TAG in plant leaves producing NoDGTT5 (Figs. 4, 5), sufficient to detect the presence of small LDs. A similar increase in LDs was seen in leaf mesophyll cells in *CrDGTT2*-expressing *Arabidopsis* [16]. Both TAG levels and LD numbers were positively correlated with *NoDGTT5* expression levels. Substantial differences in TAG content were also observed between *Arabidopsis* wild-type and NoDGTT5-producing seeds, where more than 30% higher TAG content was found (Fig. 6a). These results have important implications for the biotechnological modification of crops by increasing DGAT expression in order to produce seeds with higher oil content, seed oils with an altered TAG composition, and plants able to accumulate TAG in non-seed tissues to increase the energy density of the vegetative biomass.

## Methods

### Materials

*Nannochloropsis oceanica* strain CCMP1779 was grown in batch culture in F/2 medium as described previously [19].

*Arabidopsis* (*Arabidopsis thaliana* ecotype Columbia-2) and the T-DNA insertion mutant *tag1*-*1* (kindly provided by Prof. J. Ohlrogge) were grown as previously described [11]. Treatment with commercially available BASTA solution (Plant Media, Dublin, OH, USA) diluted 1:1000, was used for selection of transformants. Mature rosette leaves were harvested from 4-week-old transformants for lipid, molecular, and microscopic analyses. For seed collection, some plants were grown to maturity.

*Nicotiana benthamiana* plants were grown as described in [40]. Four-week-old plants were chosen for transient expression experiments.

### Sequence analysis

For the analysis of the DGATs sequence on the amino acid level, the PRofile ALIgNEment (PRALINE) software (http://www.ibi.vu.nl/programs/pralinewww/) was used [41]. Proteins used for analysis and the references are listed in Additional file 12: Table S5.

### *DGAT* expression analysis by RT-PCR

For RNA extraction, 50 ml of *N. oceanica* cultures growing in N-replete or N-deplete media was collected at 0, 3, 6, 12, 24, 48, and 72 h and three biological replicates were harvested for each time point. RNA was extracted following procedures described previously by [42]. cDNA was produced using GoScript™ Reverse Transcription System (Promega, Madison, WI, USA). Primers designed for RT-qPCR assays are given in Additional file 13: Table S1; the actin gene (NannoCCMP1779_509) was used as a reference gene. Sybr Green Master Mix (Thermo Scientific, Life Technologies Corporation, Grand Island, NY USA) and Eppendorf Mastercycler^®^ ep realplex (Eppendorf, Hauppauge, NY, USA) was used for quantitative PCR. Relative gene expression was estimated using the _ΔΔ_C_t_ method [43].

For analysis of *NoDGTT5* mRNA levels in transgenic *A. thaliana* plants, total RNA was extracted from leaves or seeds of 6-week-old transgenic and wild-type plants. cDNA synthesis, quantitative RT-PCR with gene-specific primers (Additional file 13: Table S1), and data analysis followed methods previously described [44].

### Expression of *NoDGTT1*-*NoDGTT6* in yeast

RNA from N-deprived *N. oceanica* cells was extracted, and cDNA was synthesized as described above. The primer pairs for full-length gene amplification were designed based on DGAT sequences from the *N. oceanica* CCMP1779 genome [19] and are given in Additional file 6: Table S2. PCR products were gel-purified using E.Z.N.A.^®^ Gel Extraction Kit (OMEGA Biotek) and ligated with pYES2.1 TOPO^®^ Vector (Invitrogen, Thermo Scientific, Life Technologies Corporation) according to the manufacturer’s instructions. Amplified products were sequenced by the MSU-Research Technology Support Facility in order to confirm their identity. Yeast strain H1266 (*are2D lro1D dga1D*) [24] was used for transformation, following the procedure of the pYES2.1 TOPO^®^ TA Expression Kit. After 48 h, 50 ml of the cultures was pelleted by centrifugation for 5 min at 3000*g* and collected for microscopic studies or stored in −80 °C for lipid and protein analysis.

### Immunoblotting

Proteins were extracted from H1266 transformants according to the pYES2.1 TOPO^®^ TA Yeast Expression Kit manual. Bradford protein assay was used for determination of protein concentrations with a bovine serum albumin (BSA) as a standard [45]. Protein separation and immunoblot analysis with primary His-tag antibody (GenScript, Piscataway, NJ, USA) and secondary antibody conjugated with horseradish peroxidase (Sigma Aldricht, St. Luis, MO, USA) were carried out according to [46]. Clarity™ Western ECL Substrate (Bio-Rad, Hercules, CA, USA) and ChemiDoc™ MP Imaging System (Bio-Rad) were used for detection of immunoreaction.

### DGAT microsomal activity

Isolation of microsomal fractions from strain H1266 was carried out as described in [16]. In vitro DGAT enzymatic assays were performed according to the protocol described previously by [47], with a few minor modifications. In one set of experiments, 50 µg of microsomal protein was added to a 100 µl of mix containing 50 mM MES pH 7.6, 8 mM MgCl_2_, 1 mg/ml fatty acid-free BSA, 0.25 mg C6:0-1,2-DAG (Cayman Chemical, Ann Arbor, MI, USA), and 1.8 nmol of one of the following CoA substrates: [1-^14^C]-16:0-acyl-CoA, [1-^14^C]-18:0-acyl-CoA, [1-^14^C]-18:1-acyl-CoA, [1-^14^C]-18:3-acyl-CoA, or [1-^14^C]-20:4-acyl-CoA (American Radiolabeled Chemicals Inc., St. Louis, MO, USA). The reaction was incubated at 30 °C for 30 min with shaking at 650 rpm and quenched by adding 90 µl 0.15 M acetic acid and 400 µl of chloroform: methanol (1:1 [v/v]). The organic phase was dried under N_2_ stream, dissolved in 30 µl of chloroform and spotted onto a silica TLC plate, which was developed with 80:20:1 (v/v/v) petroleum ether:ethyl ether:acetic acid. The TLC plate was exposed to film for 72 h to visualize the radiolabeled lipids. The intensity of the band corresponding to TAG was measured by using ImageJ 1.41 software. In another set of experiments (competition assay), 50 µg of microsomal protein was added to a reaction mixture composed as described above, but containing a mix of unlabelled CoA substrates: 16:0-acyl-CoA, 18:0-acyl-CoA, 18:1-acyl-CoA, and 20:4-acyl-CoA, each at final concentration of 5 µM. Lipids were extracted as described above and dissolved in 50 µl of tetrahydrofuran (THF):methanol (MeOH):water (4:4:1 [v/v/v]) and TAGs were analyzed by UPLC-nano ESI–MS/MS as described below.

### Analysis of TAG species by UPLC-nano ESI–MS/MS

UPLC-nano ESI-MS/MS molecular species analysis was performed as previously described in [48], with some modifications. The analysis was started by ultra performance liquid chromatography (UPLC) using an ACQUITY UPLC^®^ I-class system (Waters Corp., Milford, MA, USA) equipped with an ACQUITY UPLC^®^ HSS T3 column (100 mm × 1 mm, 1 μm; Waters Corp., Milford, MA, USA). Aliquots of 2 μl were injected, the flow rate was 0.13 ml/min, and the separation temperature was 35 °C. Solvent B was tetrahydrofuran/methanol/20 mM ammonium acetate (6:3:1; v/v/v) containing 0.1% (v/v) acetic acid; and solvent A was methanol/20 mM ammonium acetate (3:7; v/v) containing 0.1% (v/v) acetic acid. TAG species were separated with the following linear binary gradient: 90% solvent B held for 2 min, linear increase to 100% solvent B for 2 min, 100% solvent B held for 4 min and re-equilibration to start conditions in 4 min.

Chip-based nanoelectrospray ionization (nano ESI) was achieved with a TriVersa Nanomate^®^ (Advion, Ithaca, NY, USA) in the positive ion mode with 5-μm internal diameter nozzles. The ion source was controlled with the Advion ChipSoft Manager software. By using a post-column splitter, 330 nl/min of the eluent was directed to the nanoESI chip and ionization voltage was set to 1.41 kV. TAG molecular species were detected with a 6500 QTRAP^®^ tandem mass spectrometer (AB Sciex, Framingham, MA, USA) by monitoring the fatty acid-associated neutral loss from [M + NH_4_]^+^   molecular ions. Dwell time was 20 ms and MS parameters were optimized to maximize detector response. The integration workflow made use of the Analyst^®^ IntelliQuan (MQII) peak-finding algorithm.

### Construction of *Nannochloropsis* expression vectors

The hygromycin resistance cassette from pSELECT100 [19] and a gateway cloning site with luciferase reporter [49] were transferred to the high-copy pGEM backbone to form pNOC-Dlux. The LDSP 3′ UTR and terminator was amplified by PCR on *Nannochloropsis* genomic DNA, using primers given in Additional file 14: Table S4. The PCR product was blunt cloned with Zero Blunt^®^ PCR Cloning Kit (Invitrogen, ThermoFisher Scientific), sequenced and transferred to the *Sac*I and *Afl*II sites in the pNoc-Dlux plasmid. The elongation factor (EF) promoter was amplified by PCR from *Nannochloropsis* genomic DNA (primers given in Additional file 14: Table S4) and inserted in the pENTR gateway entry vector by using pENTR™/D-TOPO^®^ Cloning Kit (Invitrogen, ThermoFisher Scientific), sequenced, and transferred to pNoc-Dlux-LDSP terminator by a LR clonase reaction (Invitrogen). The luciferase reporter was removed by digestion with *Asc*I and *Sac*I and replaced with venus fluorescent protein (Additional file 9: Figure S8A) or green fluorescent protein (Additional file 9: Figure S8B) genes, amplified by PCR with the primers given in Additional file 14: Table S4, blunt cloned as described above, sequenced and inserted into the *Hpa*I and *Mlu*I sites.

### Expression of *NoDGTT5* in *N. oceanica* CCMP1779

The full sequence of *NoDGTT5* was amplified as described above using forward and reverse primers given in Additional file 15: Table S3. The amplified product of 1092 bp was gel-purified by using E.Z.N.A. Gel Extraction Kit (OMEGA Biotek) and ligated to the *pnoc ox venus* vector (Additional file 9: Figure S8) using the protocol for T4 DNA Ligase (New England BioLabs^®^Inc, USA). Amplified constructs were sequenced by the MSU-Research Technology Support Facility in order to confirm their identity. Nuclear transformation of *N. oceanica* CCMP1779 cells was carried out as described in [19]. Expression of NoDGTT5 under its native promoter was carried out as described above using the *pnoc gfp dggt5pro* vector (Additional file 13: Figure S8B).

### Transient expression of *NoDGTT5* in *Nicotiana benthamiana* leaves

*Agrobacterium tumefaciens*-mediated transient expression of the full-length *NoDGTT5* in *N. benthamiana* leaves was performed as described previously by [40].

### Transformation of Arabidopsis plants

35S:YFP-NoDGTT5 construct was generated using the GATEWAY system (https://www.lifetechnologies.com/us/en/home/life-science/cloning/gateway-cloning.html) by subcloning the full coding sequence of NoDGTT5 into pEarleyGate 101 vector tagged with YFP at the C-terminus. Stem and flowers of Col-2 and *tag1*-*1* were dipped into an inoculation solution (OD_600_ = 0.8; 5% (w/v) sucrose and 0.005% (v/v) silwet-77) containing *A. tumefaciens* as described in [50]. The plants were incubated in the dark overnight and afterwards grown under long day conditions. Selected transformants where grown for 4 weeks under conditions described above; mature rosette leaves were harvested for lipid analysis, DNA and RNA extraction and for microscopic studies.

### Lipid extraction and analysis

For each sample of *N. oceanica* CCMP1779 total lipids were extracted from cell pellets obtained after centrifugation of 50 ml of culture (20 × 10^6^ cells/ml). Lipid extraction and separation were performed as described in [42]. FAME preparation and quantification were carried out according to [19].

Positional analysis of TAG was performed with *R. arrhizus* lipase according to the procedure previously described in [51].

Lipids from H1226 yeast transformants were extracted based on a protocol described by [52]. Lipids separation by TLC, FAME reaction and TAG content estimation (as a percentage of total lipids) was carried out as described in [16].

Total lipid extraction and estimation of TAG content from were carried out as described in [53] for Arabidopsis leaves and as described in [54] for Arabidopsis seeds. Lipids from tobacco leaves were extracted and TAGs were separated as described in [55].

### Acyl-CoA profiling

Triplicates of 50 mg of fresh non-transformed Arabidopsis and tobacco leaves and 50 mg of *N. oceanica* CCMP1779 cells resulting from centrifugation of liquid culture growing under N replete conditions were used for acyl CoAs extraction. Acyl-CoAs were extracted as described by [56]. Briefly, each sample was homogenized in 200 μl of freshly prepared extraction buffer [2 ml 2-propanol, 2 ml pH 7.2 50 mM KH_2_PO_4_, 50 μl glacial acetic acid, 80 μl 50 mg/ml fatty acid-free BSA]. Lipids and pigments were removed by washing the extract three times with 300 μl petroleum ether and saturated with 2-propanol:water (1:1; v/v). Between washes, the phases were separated by low-speed centrifugation (500*g*) for 2 min. The upper phases were discarded. Following the petroleum ether washes, 5 μl saturated (NH_4_)_2_SO_4_ was added to the extract followed by 600 μl methanol:chloroform (2:1; v/v). After vortexing, the samples were incubated for 20 min at room temperature and centrifuged at 21,000*g* for 5 min. The supernatant was carefully collected, dried under streaming nitrogen and reconstituted in 100 μl of derivatizing reagent [3.165 ml chloroacetaldehyde + 250 mg SDS + 46.835 ml of citrate buffer pH 4.0]. After 20 min of derivatization at 85 °C, the samples were cooled down and centrifuged at maximum speed for 5 min. The samples were processed and analyzed by using HPLC as described in [56].

### Seed imaging and morphometrics

Dry seeds of Arabidopsis were briefly washed in 96% ethanol and mounted on glass slides. The samples were observed under a Leica M60 Stereo Microscope equipped with a camera. Mean length and width (μm) of seeds were measured and analyzed using ImageJ 1.41 software.

### Confocal microscopy

For lipid droplets, visualization in living H1266 cells and Arabidopsis leaves BODIPY 493/503 (ThermoFisher Scientific) at a final concentration of 10 μg/ml in PBS buffer was used. The staining was carried out by 1 h incubation at room temperature and followed by two washes in PBS. Samples were then immediately observed in Olympus Spectral FV1000 microscope (Olympus, Japan) using an argon (488 nm) laser.

YFP/VFP detection in transformed *N. oceanica* CCMP1779 and tobacco leaves was analyzed with Olympus Spectral FV1000 microscope (Olympus, Japan) at the excitation wavelength of 515 nm. Chloroplast autofluorescence was excited with a solid state laser at 556 nm. All CLSM figures represent Z-series images composed using the Olympus FluoView FV1000 confocal microscope software (Olympus). Negative controls were treated as above, but the dye was omitted.

### Transmission electron microcopy

H1266 cells were fixed in 5% (v/v) glutaraldehyde, post-fixed in 1% OsO_4_ and embedded in Spurr’s resin (Electron Microscopy Sciences, Hatfield, PA, USA) following the methods described by [57]. Ultrathin sections (70 nm thick) were cut on a RMC MYX ultramicrotome (Boeckeler Instruments, Inc. Tucson, AZ, USA) and mounted on 150 mesh formvar-coated copper grids.

For TEM analysis, Arabidopsis seeds were imbibed in water for 3 h at room temperature and the seed coat was removed manually from each seed. The seeds were then fixed and processed as described above.

Images were taken with a JEOL100 CXII transmission electron microscope (Japan Electron Optics Laboratories, http://www.jeol.co.jp).

## Additional files

**Additional file 1: Figure S1.** Sequence alignment of type 1 DGATs from *N. oceanica* CCMP 1779 (NoDGAT1), *Arabidopsis* (AtDGAT1), human (HsDGAT1), mouse (MmDGAT1) and rat (RnDGAT1).

**Additional file 2: Figure S2.** Identified conserved sequence motifs in type 1 DGATs from *N. oceanica* CCMP1779 (NoDGAT1), *Arabidopsis* (AtDGAT1), human (HsDGAT1), mouse (MmDGAT1) and rat (RnDGAT1). ∆ marks amino acids conserved among all analyzed DGATs. In B black * marks amino acids which are known to be conserved in type 1 DGATs and are also conserved in *N. oceanica* DGAT1. A red * marks amino acids known to be conserved in DGATs of type 1, but are not conserved in *N. oceanica* DGAT1.

**Additional file 3: Figure S3.** Sequence alignment between type 2 DGATs (DGTTs) of *N. oceanica* CCMP1779.

**Additional file 4: Figure S4.** Conserved sequence motifs of type 2 DGATs identified of *N. oceanica* CCMP1779 DGTTs, *Arabidopsis* (AtDGAT2) and human (HsDGAT2). A red Δ indicates the amino acid, which is not totally conserved among *N. oceanica* CCMP1779 DGTTs. A red * marks amino acids which are known to be conserved in DGATs of type 2, but not conserved in *N. oceanica* CCMP1779 DGTTs.

**Additional file 5: Figure S5.** Sequence conservation of YFP motif and of flanking regions of the PH motif in type 2 DGATs of *N. oceanica* CCMP1779 (NoDGTTs), *Arabidopsis* (AtDGAT2) and human (HsDGAT2). The multiple sequence alignment was generated using the Praline software. Amino acid residues are color-coded according to their hydrophobicity (legend at the bottom of the figure). The notation in brackets indicates omitted amino acids.

**Additional file 6: Table S2.** Primers used for amplification of *NoDGTT1*-*NoDGTT6* full sequences for cloning into pYES2.1/V5-His-TOPO® vector.

**Additional file 7: Figure S6.** (A) Confirmation of NoDGTT1-6 protein expression in yeast by immunoblotting analysis. (B) CLSM analysis of LDs formation in H1266 strains expressing *NoDGTT1*-*NoDGTT4* and *NoDGTT6*. Bars = 5 μm (C) Ultrastructural analysis of non-transformed H1266 and transgenic yeast by TEM. Lipid droplets (arrowheads) are present in *NoDGTT3-* and *NoDGTT5* over-expressing lines; V, vacuole. Bars = 0.5 μm.

**Additional file 8: Figure S7.** (A–B) NoDGTT5 activity in H1266 microsomes. (A) Autoradiograph of radiolabeled lipids separated on a TLC plate after reaction with distinct CoA substrates. Signals corresponding to TAG, free fatty acids (FFA) and diacylglycerol (DAG) are indicated. H1266+NoDGTT5 and H1266+EV indicate microsomes from NoDGTT5 and control vector expressing yeast, respectively. (B) Densytometric data corresponding to the TAG band of H1266+NoDGTT5 from (A). (C) Composition of acyl-CoA pools in *N. oceanica* CCMP1779 growing under N replete conditions and in leaves of wild type *Arabidopsis* and tobacco. The graphs show the relative content of acyl chains of diverse species in a total pool of acyl-CoAs. If the chromatographic signals of the acyl CoA species were not baseline separated the mixed composition of the respective signals is indicated. The data represent mean values of 3 biological replicates. Error bars indicate standard deviation.

**Additional file 9: Figure S8.** Vectors used in the present study for over-expression and expression of *NoDGTT5* in *N. oceanica* CCMP1779 under control of EF promoter (A) and its native promoter (B). AmpR; ampicillin resistance cassette, LDSP; lipid droplet surface protein, HygR; hygromycin B resistance cassette, EF; elongation factor, GFP; green fluorescence protein, NOS; nopaline synthase.

**Additional file 10: Figure S9.** (A) Immunoblot to the venus protein (anti-GFP Ab) in lines over-expressing *NoDGTT5* under control of EF promoter (B) CLSM analysis of control (top panel) and positive (bottom panel) transformants for the presence of venus fluorescent protein (VFP). In each image, insets correspond to zoomed area marked with the dashed line. Bars = 5 μm. (C) CLSM analysis of control (top panel) and empty vector (bottom panel) transformants for the presence of GFP fluorescence. Bars = 5 μm.

**Additional file 11: Figure S10.** (A–B) Relative expression of *NoDGTT5* analyzed by qPCR in the leaves (A) and seeds (B) of *Arabidopsis* lines transformed with 35S:NoDGTT5 construct. (C) Analysis for the insertion of T-DNA by PCR using genomic DNA from the wild-type plants (AtWT), control lines (AtEV) and two *tag1-1* lines (*tag1-1*a and *tag1-1*b). (D) Relative expression of *NoDGTT5* analyzed by qPCR in the seeds of two *tag1-1* lines complemented with *NoDGTT5*. In A and B arrowheads indicate the lines exhibiting the highest *NoDGTT5* expression and chosen for lipid and microscopic analyses. Each data point represents the average of three biological replicates. Each sample was analyzed in technical triplicates and normalized to actin as a reference gene. Values represent means ± SD (*n* = 3), with asterisks indicating *P* < 0.01.

**Additional file 12: Table S5.** Information on DGATs sequences used for analysis with PRALINE software.

**Additional file 13: Table S1.** Primers used for reverse transcription quantitative PCR.

**Additional file 14: Table S4.** Primers used for amplification of sequences of *pnoc ox venus* vector and *pnoc gfp dgtt5pro* vector used for transformation of *N. oceanica* CCMP1779.

**Additional file 15: Table S3.** Primers used for amplification of sequences of listed genes without stop codon for cloning into pEarleyGate 101 vector. DGTT5 PRO refers to *NoDGTT5* sequence including its native promoter.

## Abbreviations

ARE2: acyl-coenzyme A: cholesterol acyl transferase 2
CLSM: confocal scanning laser microscopy
CoA: coenzyme A
DAG: diacylglycerol
DGA1: diacylglycerol acyltransferase 1
DGAT: acyl-CoA:diacylglycerol acyltransferase of type 1
DGTT: acyl-CoA:diacylglycerol acyltransferase of type 2
EF: elongation factor
ER: endoplasmic reticulum
FA: fatty acid
FFA: free fatty acids
FAME: fatty acid methyl ester
GC-MS: gas chromatography-mass spectrometry
GFP: green fluorescent protein
LD: lipid droplet
LRO1: lecithin:cholesterol acyl transferase 1
PC: phosphatidylcholine
PDAT: phospholipid:diacylglycerol acyltransferase
TAG: triacylglycerols
TEM: transmission electron microscopy
TLC: thin-layer chromatography
VFP: venus fluorescent protein
YFP: yellow fluorescent protein
