# Supplementary figures and images for: Nannochloropsis, a rich source of diacylglycerol acyltransferases for engineering of triacylglycerol content in different hosts
Source: Biotechnol Biofuels. 2017 Jan 3;10:8. doi: 10.1186/s13068-016-0686-8 (PMC5210179; doi:10.1186/s13068-016-0686-8)

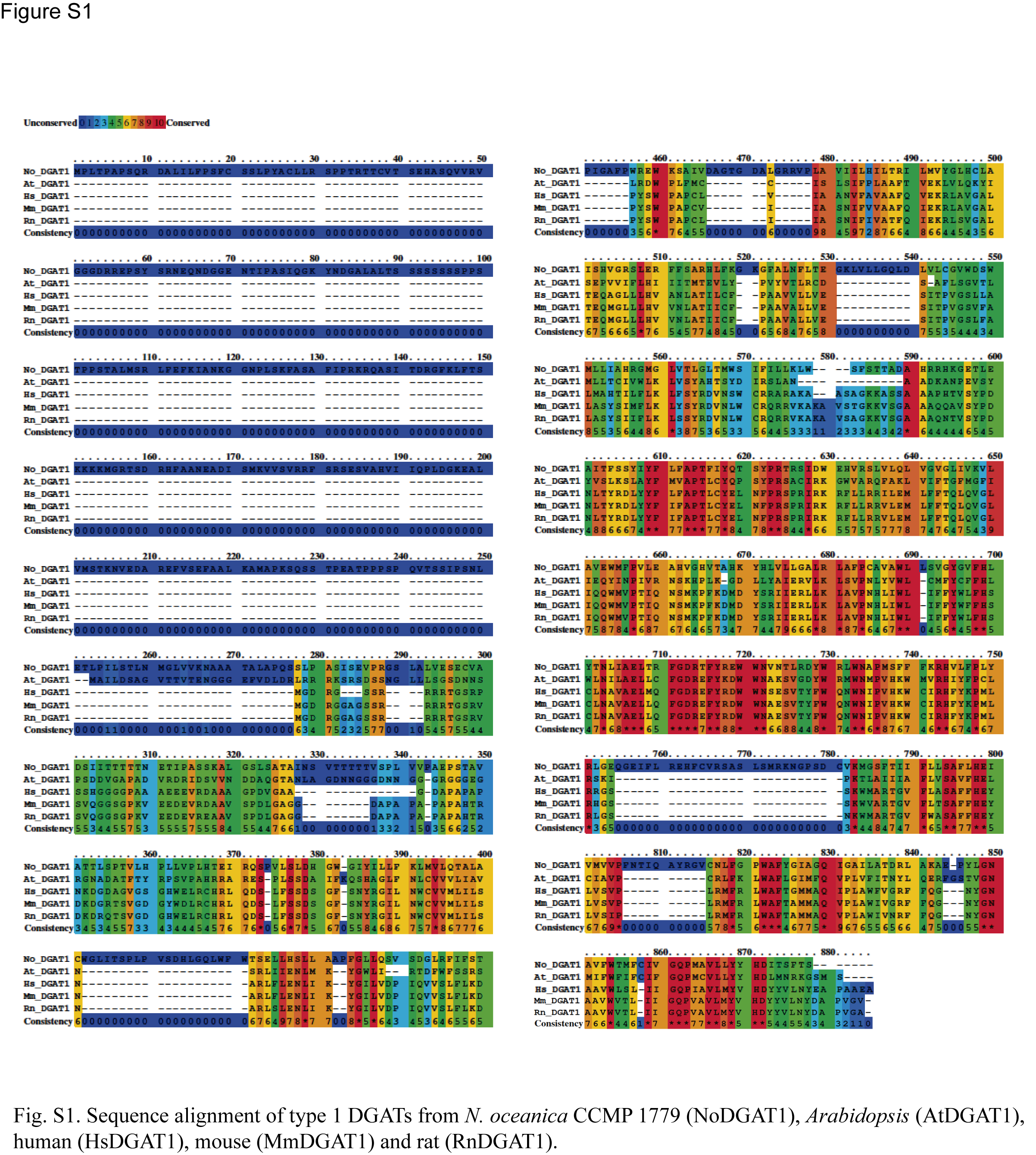

Supplement: Supplementary file 1 — Additional file 1: Figure S1. Sequence alignment of type 1 DGATs from N. oceanica CCMP 1779 (NoDGAT1), Arabidopsis (AtDGAT1), human (HsDGAT1), mouse (MmDGAT1) and rat (RnDGAT1). [file 13068_2016_686_MOESM1_ESM.tif]

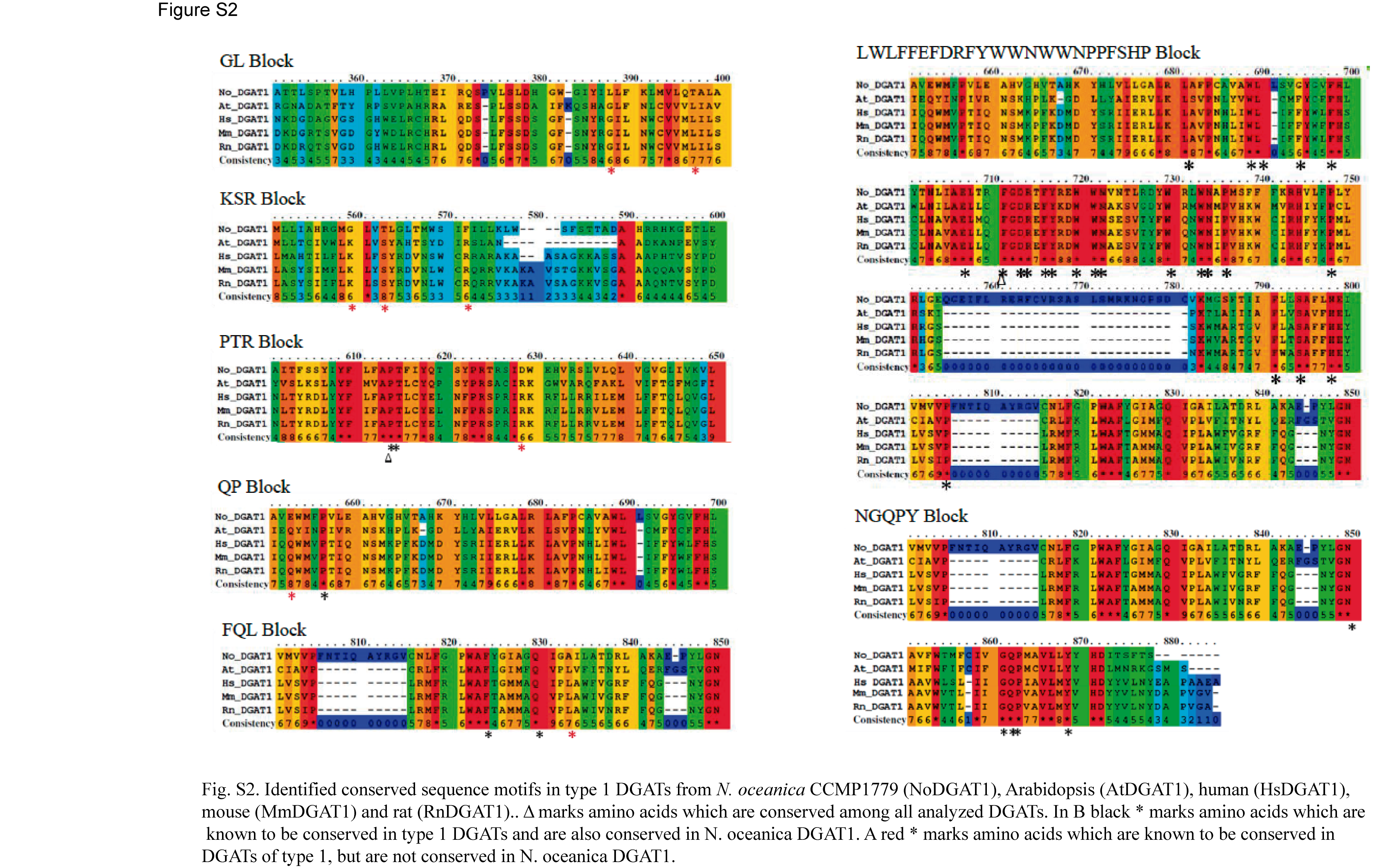

Supplement: Supplementary file 2 — Additional file 2: Figure S2. Identified conserved sequence motifs in type 1 DGATs from N. oceanica CCMP1779 (NoDGAT1), Arabidopsis (AtDGAT1), human (HsDGAT1), mouse (MmDGAT1) and rat (RnDGAT1). ∆ marks amino acids conserved among all analyzed DGATs. In B black * marks amino acids which are known to be conserved in type 1 DGATs and are also conserved in N. oceanica DGAT1. A red * marks amino acids known to be conserved in DGATs of type 1, but are not conserved in N. oceanica DGAT1. [file 13068_2016_686_MOESM2_ESM.tif]

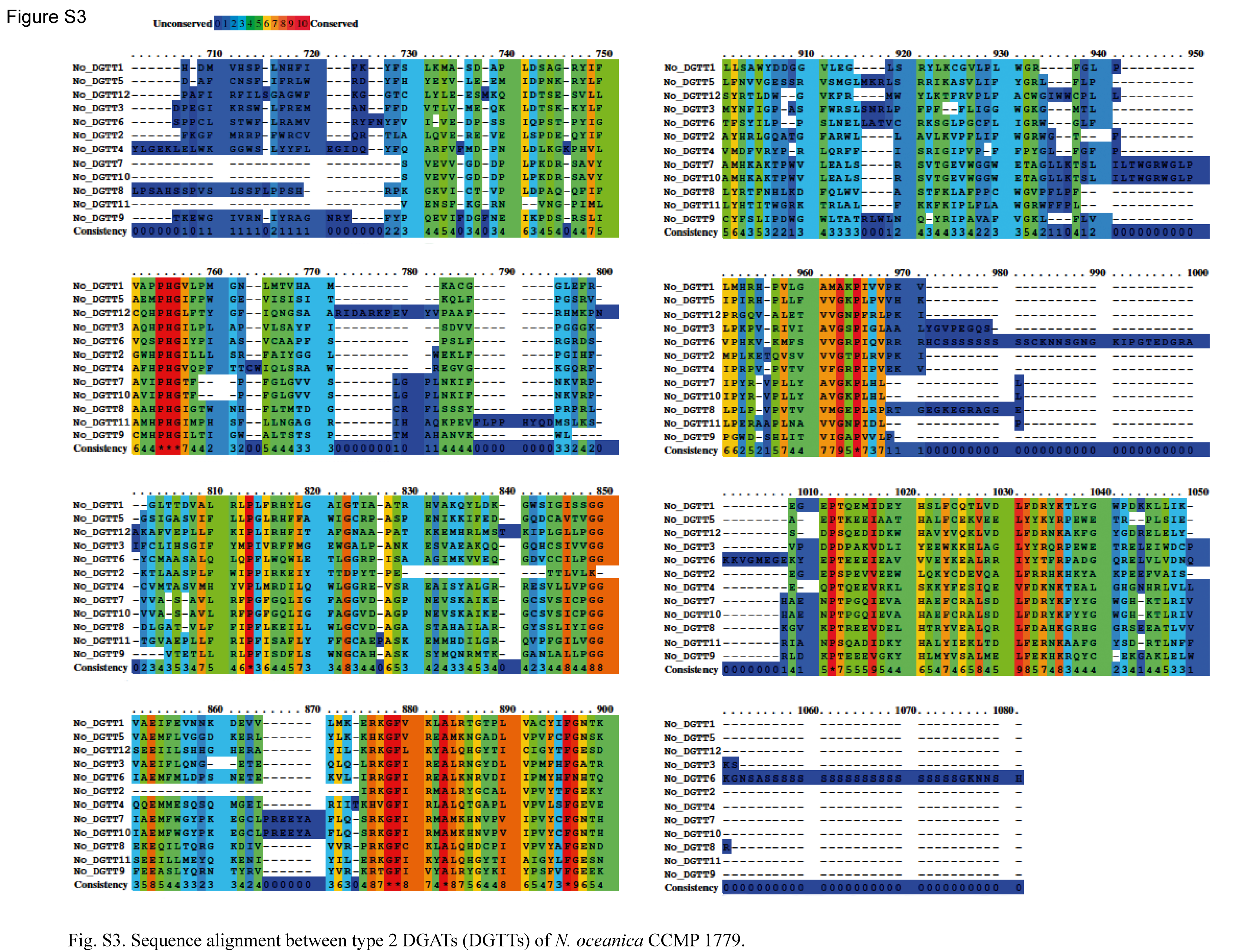

Supplement: Supplementary file 3 — Additional file 3: Figure S3. Sequence alignment between type 2 DGATs (DGTTs) of N. oceanica CCMP1779. [file 13068_2016_686_MOESM3_ESM.tif]

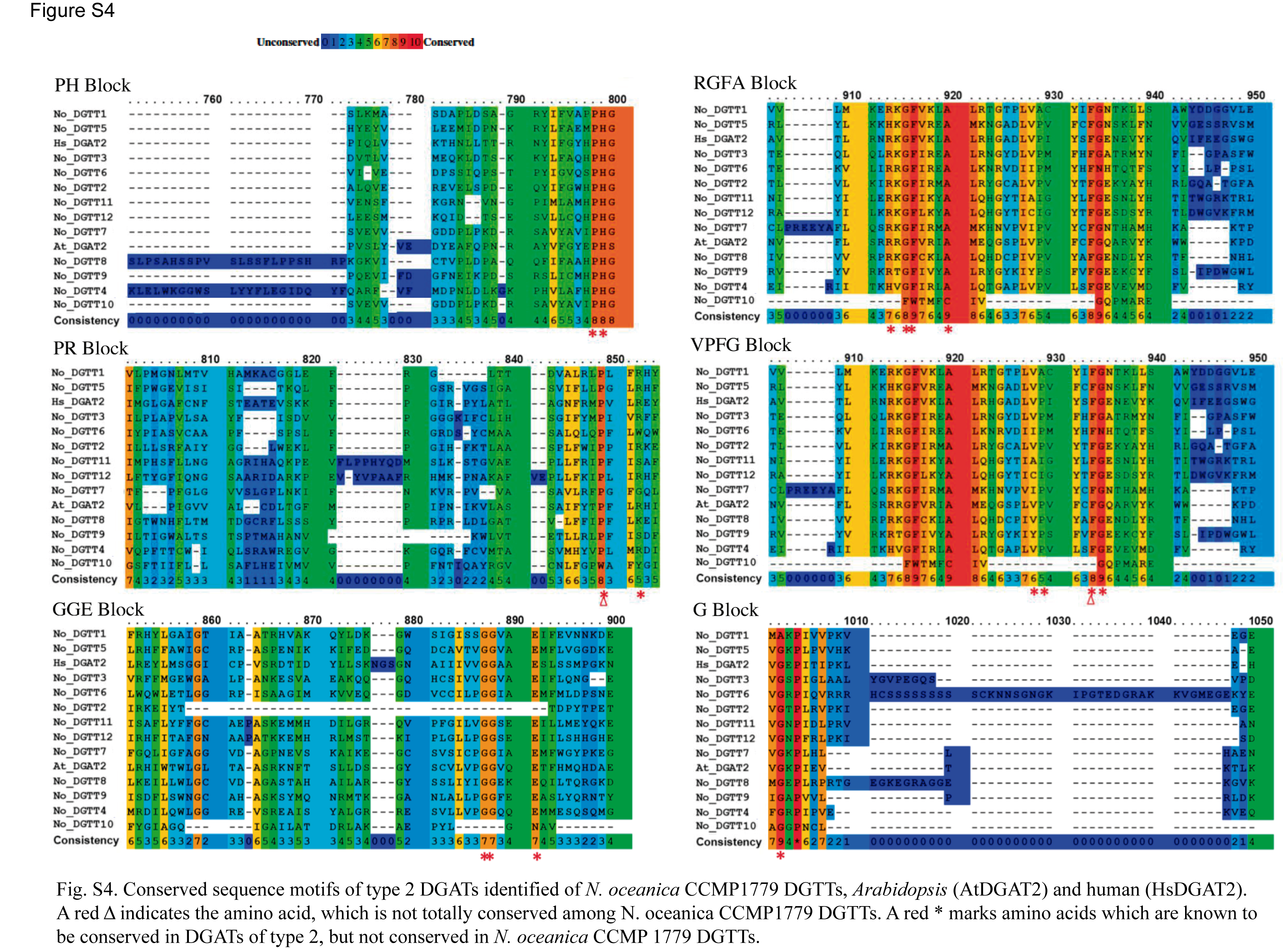

Supplement: Supplementary file 4 — Additional file 4: Figure S4. Conserved sequence motifs of type 2 DGATs identified of N. oceanica CCMP1779 DGTTs, Arabidopsis (AtDGAT2) and human (HsDGAT2). A red Δ indicates the amino acid, which is not totally conserved among N. oceanica CCMP1779 DGTTs. A red * marks amino acids which are known to be conserved in DGATs of type 2, but not conserved in N. oceanica CCMP1779 DGTTs. [file 13068_2016_686_MOESM4_ESM.tif]

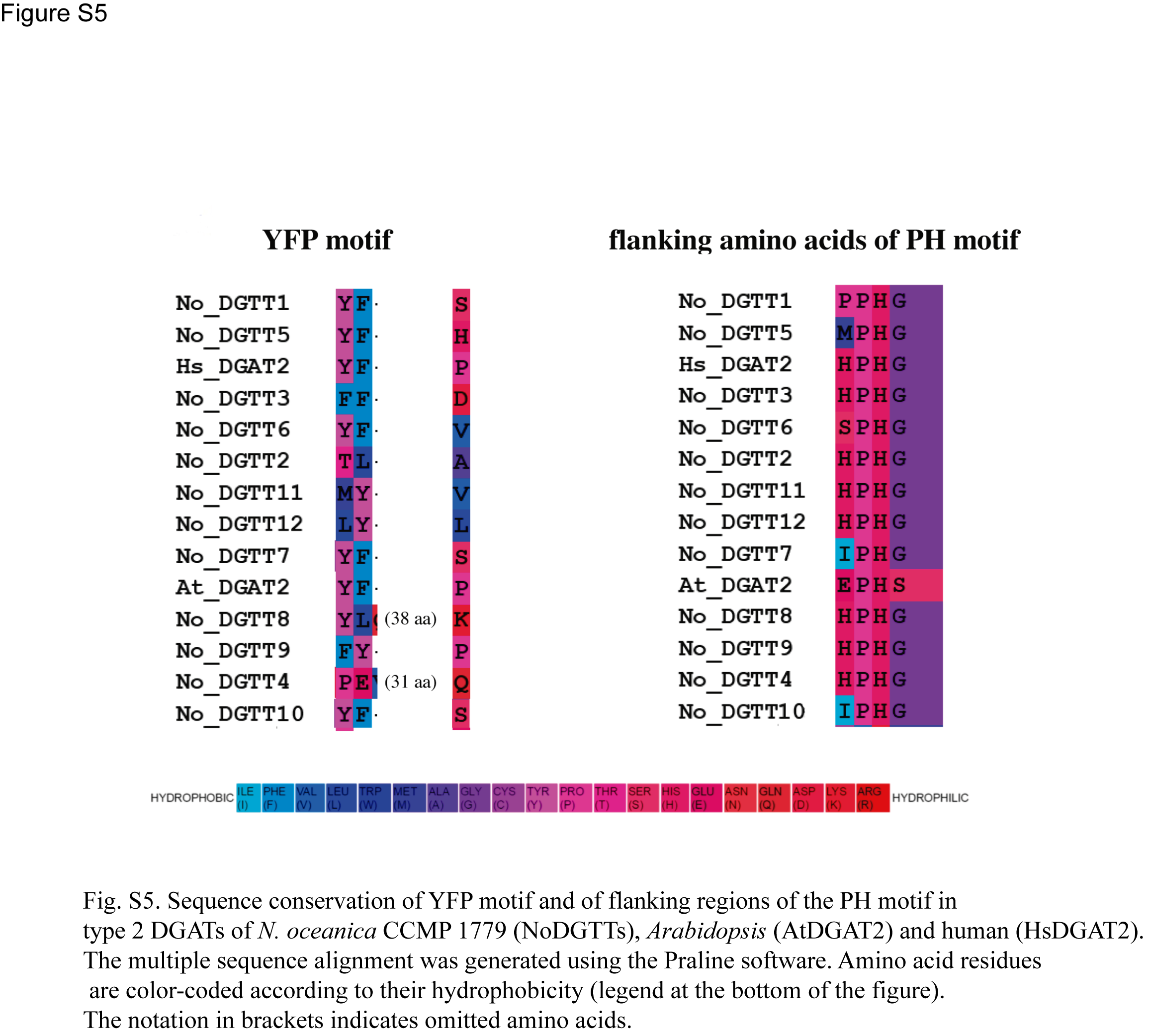

Supplement: Supplementary file 5 — Additional file 5: Figure S5. Sequence conservation of YFP motif and of flanking regions of the PH motif in type 2 DGATs of N. oceanica CCMP1779 (NoDGTTs), Arabidopsis (AtDGAT2) and human (HsDGAT2). The multiple sequence alignment was generated using the Praline software. Amino acid residues are color-coded according to their hydrophobicity (legend at the bottom of the figure). The notation in brackets indicates omitted amino acids. [file 13068_2016_686_MOESM5_ESM.tif]

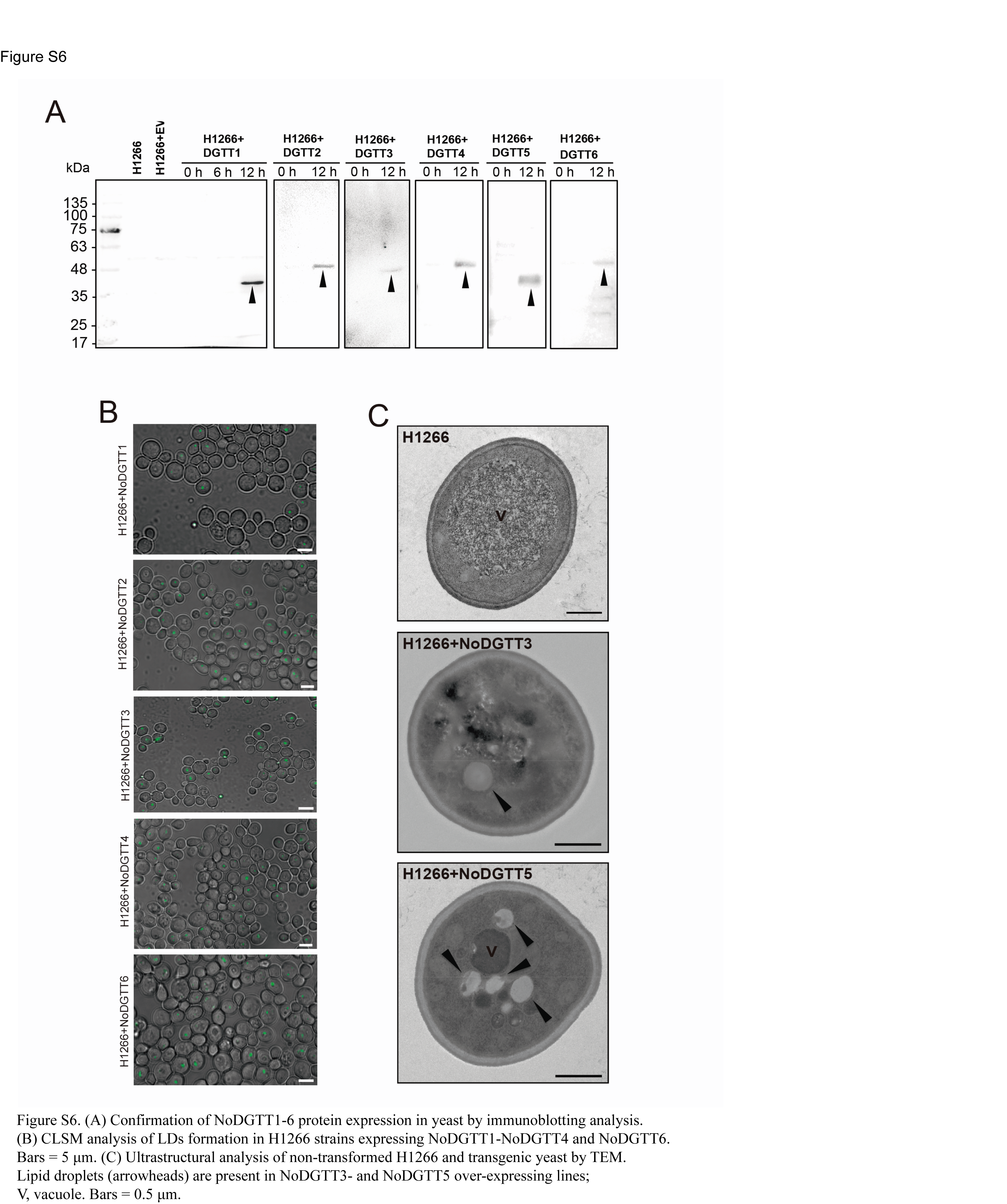

Supplement: Supplementary file 7 — Additional file 7: Figure S6. (A) Confirmation of NoDGTT1-6 protein expression in yeast by immunoblotting analysis. (B) CLSM analysis of LDs formation in H1266 strains expressing NoDGTT1-NoDGTT4 and NoDGTT6. Bars = 5 μm (C) Ultrastructural analysis of non-transformed H1266 and transgenic yeast by TEM. Lipid droplets (arrowheads) are present in NoDGTT3- and NoDGTT5 over-expressing lines; V, vacuole. Bars = 0.5 μm. [file 13068_2016_686_MOESM7_ESM.tif]

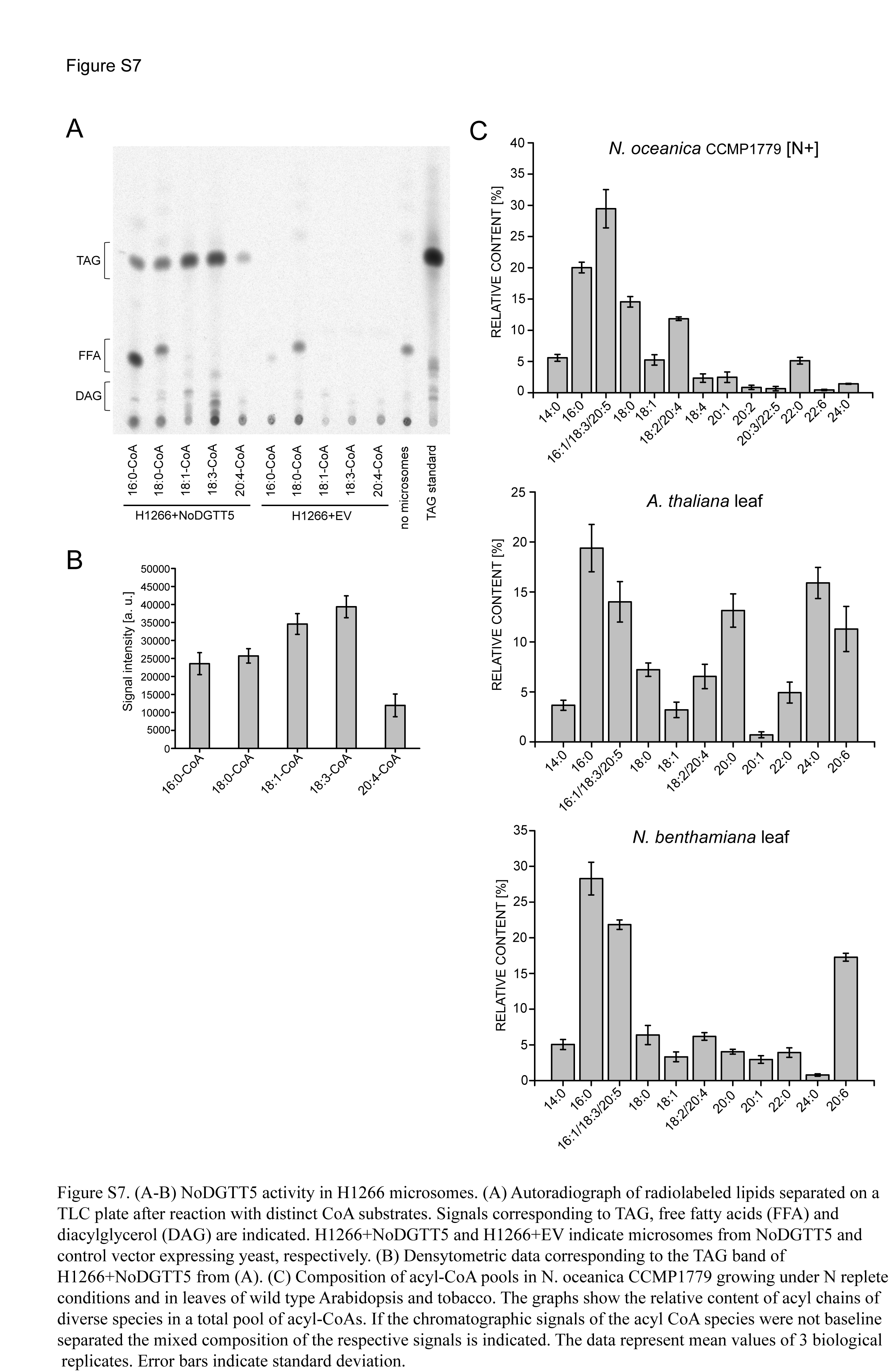

Supplement: Supplementary file 8 — Additional file 8: Figure S7. (A–B) NoDGTT5 activity in H1266 microsomes. (A) Autoradiograph of radiolabeled lipids separated on a TLC plate after reaction with distinct CoA substrates. Signals corresponding to TAG, free fatty acids (FFA) and diacylglycerol (DAG) are indicated. H1266+NoDGTT5 and H1266+EV indicate microsomes from NoDGTT5 and control vector expressing yeast, respectively. (B) Densytometric data corresponding to the TAG band of H1266+NoDGTT5 from (A). (C) Composition of acyl-CoA pools in N. oceanica CCMP1779 growing under N replete conditions and in leaves of wild type Arabidopsis and tobacco. The graphs show the relative content of acyl chains of diverse species in a total pool of acyl-CoAs. If the chromatographic signals of the acyl CoA species were not baseline separated the mixed composition of the respective signals is indicated. The data represent mean values of 3 biological replicates. Error bars indicate standard deviation. [file 13068_2016_686_MOESM8_ESM.tif]

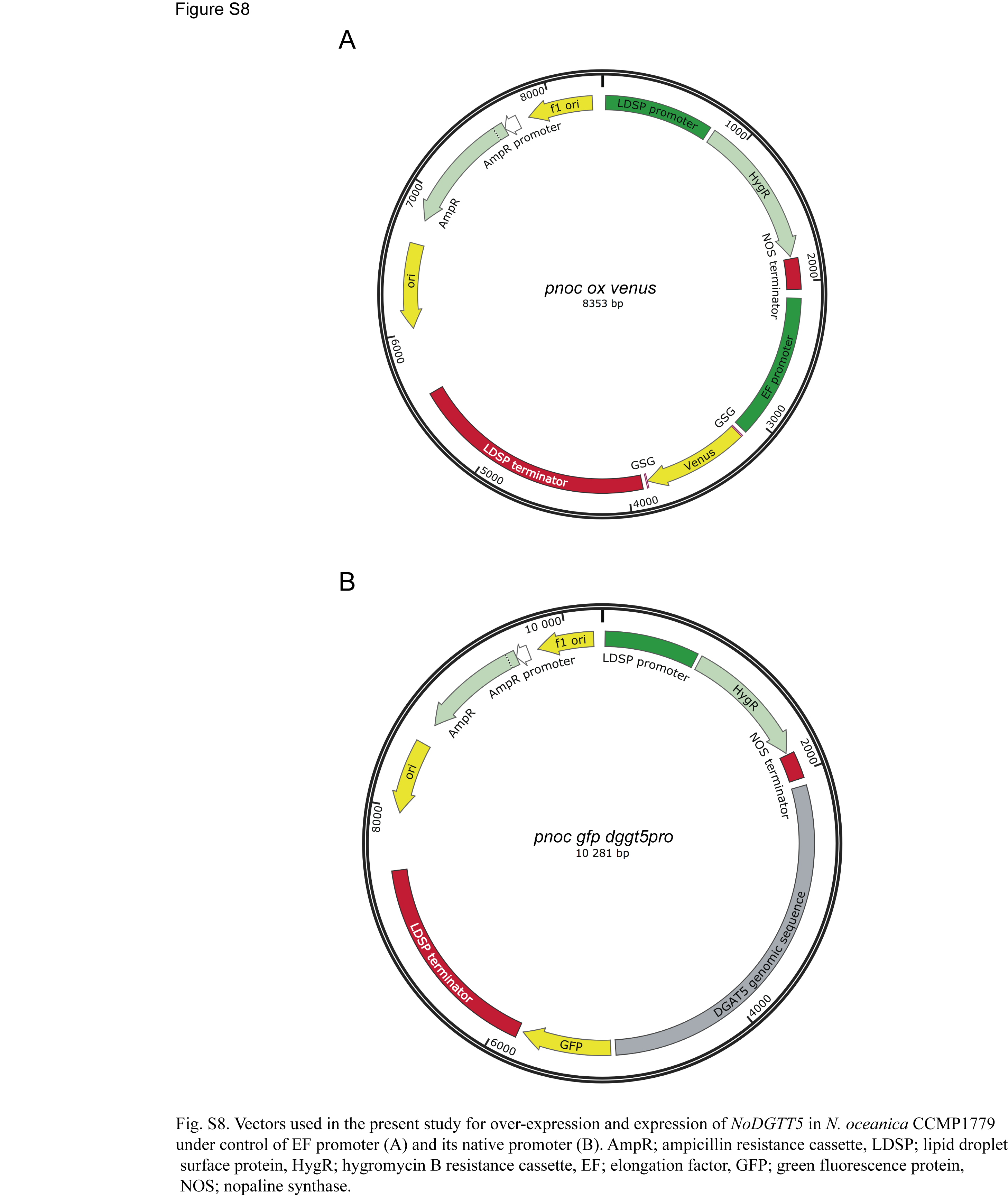

Supplement: Supplementary file 9 — Additional file 9: Figure S8. Vectors used in the present study for over-expression and expression of NoDGTT5 in N. oceanica CCMP1779 under control of EF promoter (A) and its native promoter (B). AmpR; ampicillin resistance cassette, LDSP; lipid droplet surface protein, HygR; hygromycin B resistance cassette, EF; elongation factor, GFP; green fluorescence protein, NOS; nopaline synthase. [file 13068_2016_686_MOESM9_ESM.tif]

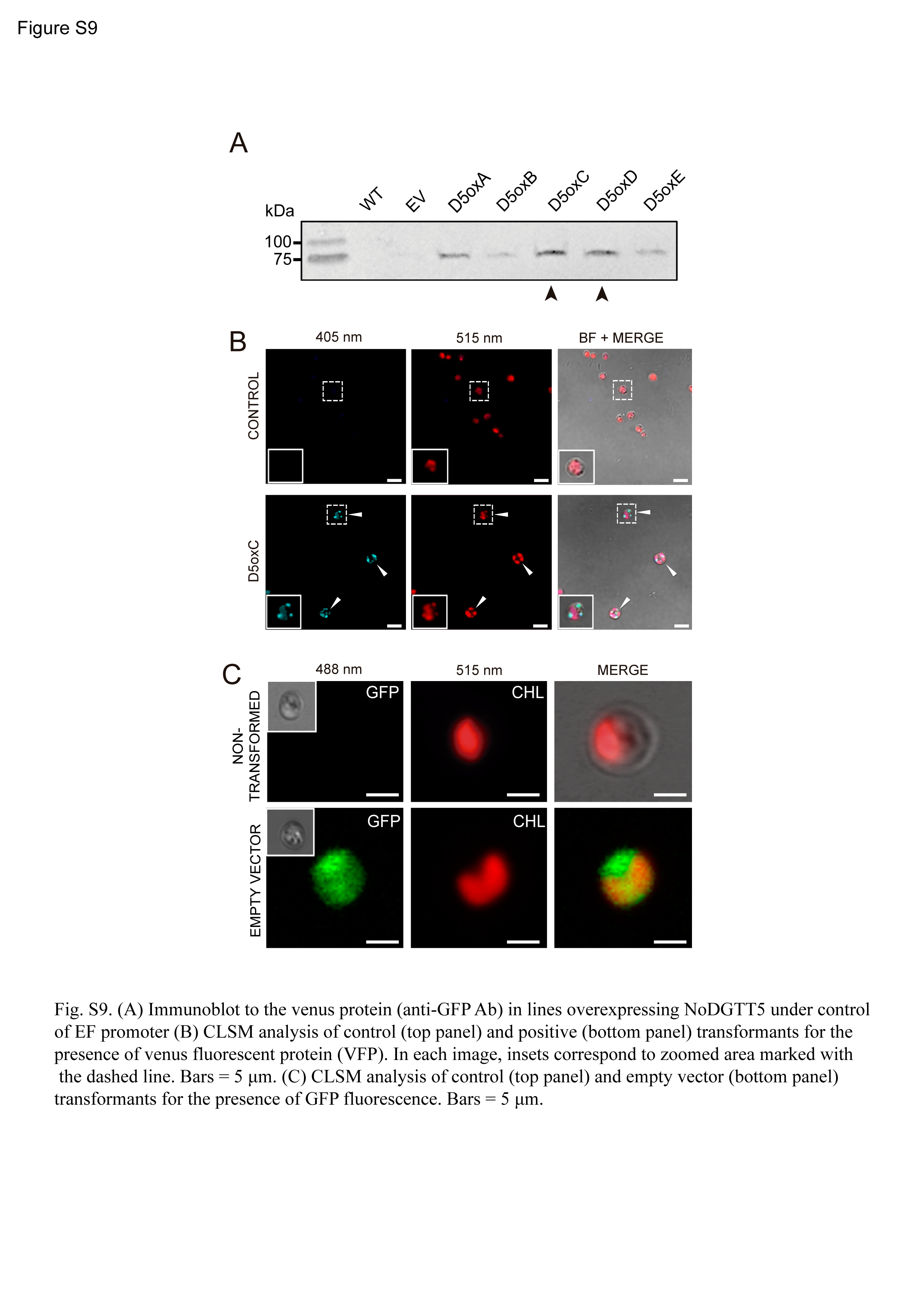

Supplement: Supplementary file 10 — Additional file 10: Figure S9. (A) Immunoblot to the venus protein (anti-GFP Ab) in lines over-expressing NoDGTT5 under control of EF promoter (B) CLSM analysis of control (top panel) and positive (bottom panel) transformants for the presence of venus fluorescent protein (VFP). In each image, insets correspond to zoomed area marked with the dashed line. Bars = 5 μm. (C) CLSM analysis of control (top panel) and empty vector (bottom panel) transformants for the presence of GFP fluorescence. Bars = 5 μm. [file 13068_2016_686_MOESM10_ESM.tif]

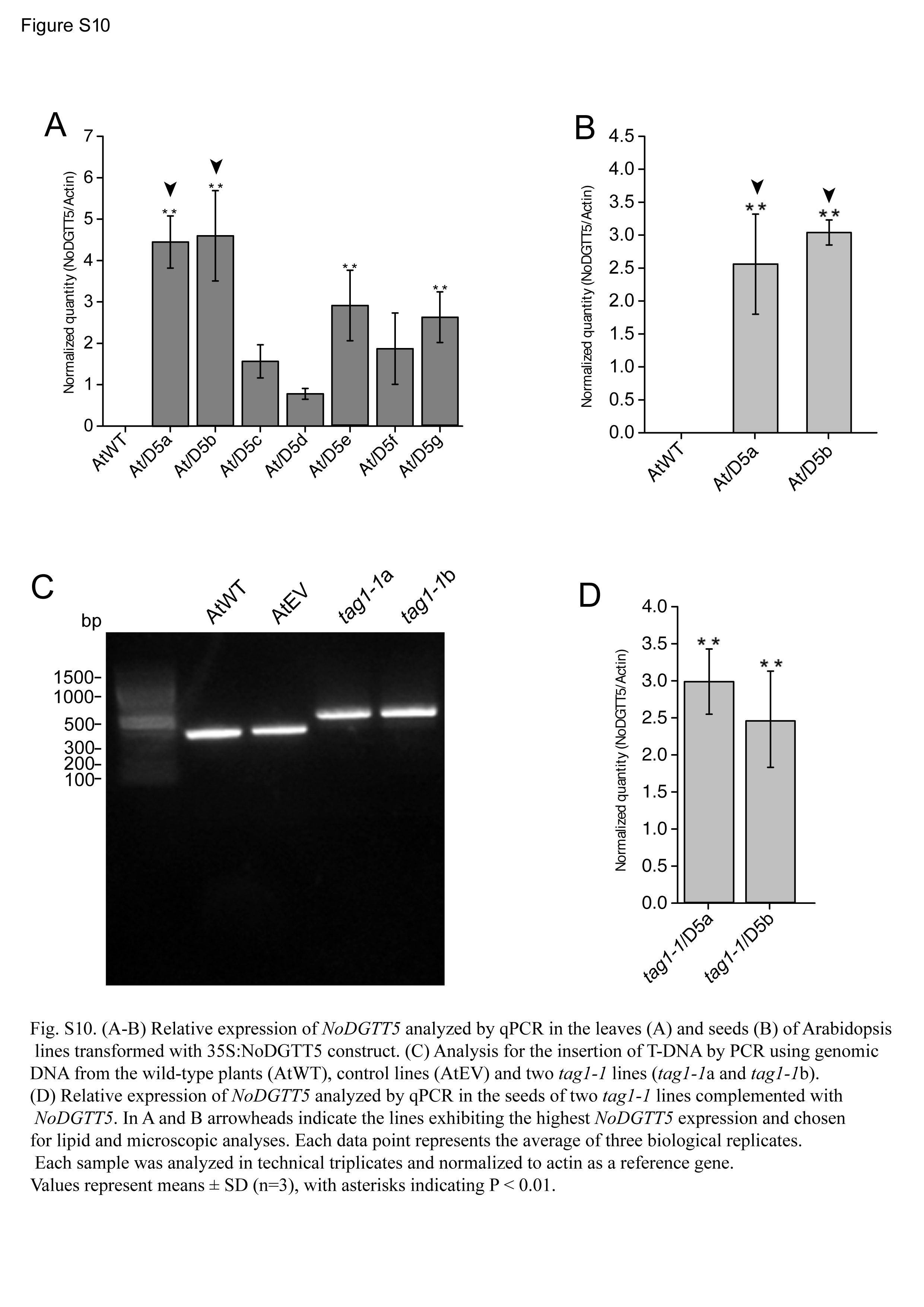

Supplement: Supplementary file 11 — Additional file 11: Figure S10. (A–B) Relative expression of NoDGTT5 analyzed by qPCR in the leaves (A) and seeds (B) of Arabidopsis lines transformed with 35S:NoDGTT5 construct. (C) Analysis for the insertion of T-DNA by PCR using genomic DNA from the wild-type plants (AtWT), control lines (AtEV) and two tag1-1 lines (tag1-1a and tag1-1b). (D) Relative expression of NoDGTT5 analyzed by qPCR in the seeds of two tag1-1 lines complemented with NoDGTT5. In A and B arrowheads indicate the lines exhibiting the highest NoDGTT5 expression and chosen for lipid and microscopic analyses. Each data point represents the average of three biological replicates. Each sample was analyzed in technical triplicates and normalized to actin as a reference gene. Values represent means ± SD (n = 3), with asterisks indicating P < 0.01. [file 13068_2016_686_MOESM11_ESM.tif]
