# Supplementary material for: Nannochloropsis, a rich source of diacylglycerol acyltransferases for engineering of triacylglycerol content in different hosts
Source: Biotechnol Biofuels. 2017 Jan 3;10:8. doi: 10.1186/s13068-016-0686-8 (PMC5210179; doi:10.1186/s13068-016-0686-8)
Supplement: Supplementary file 6 — Additional file 6: Table S2. Primers used for amplification of NoDGTT1-NoDGTT6 full sequences for cloning into pYES2.1/V5-His-TOPO® vector. [file 13068_2016_686_MOESM6_ESM.pdf]

GENE NAME	GENE ID	GenBank ACCESSION NUMBER	(5')FORWARD PRIMER(3')	(5')REVERSE PRIMER(3')
NoDGTT1	CCMP1779_4340	KY273668	ATGTACCCAATCAAGCTGTGCTTCCTC	TCACTTAATAAGCAGCTTCTTGTCCG
NoDGTT2	CCMP1779_3705	KY273669	ATGGCTCACCTCTTCCGTCG	AGAGATCGCAACGAACTCCTCG
NoDGTT3	CCMP1779_7206	KY273670	ATGGGCGCTACCACCGAGACCCA GACTAAA	CGACTTCGGACAGTCCCAAATCTC CAACTCTCGCGT
NoDGTT4	CCMP1779_9929	KY273671	ATGAAGCGGCGGCGCAGA	GCAGCGTCGCGACTGGTCT
NoDGTT5	CCMP1779_3915	KY273672	ATGACGCCGCAAGCCGACATCACC AGCAAGACGA	CTCAATGGACAACGGGCGCGTCTC CCACTCC
NoDGTT6	CCMP1779_9590	KY273673	ATGTCCTCCTTCTTGCGTTGGC	GCTATTATTCTTACCGCTGCTACTGC

Table S2. Primers used for amplification of *NoDGTT1*-*NoDGTT6* full sequences for cloning into pYES2.1/V5-His-TOPO® vector.
