# Supplementary material for: Nannochloropsis, a rich source of diacylglycerol acyltransferases for engineering of triacylglycerol content in different hosts
Source: Biotechnol Biofuels. 2017 Jan 3;10:8. doi: 10.1186/s13068-016-0686-8 (PMC5210179; doi:10.1186/s13068-016-0686-8)
Supplement: Supplementary file 12 — Additional file 12: Table S5. Information on DGATs sequences used for analysis with PRALINE software. [file 13068_2016_686_MOESM12_ESM.pdf]

PHYLOGENETIC CLASS	ORGANISM	DGAT GENE	SEQUENCE REFERENCE	LITERATURE REFERENCE
eustigmatophyceae (algae)	*Nannochloropsis oceanica* CMP1779	*NoDGAT1*	NannoCCMP1779_3520	[1]
		NoDGTT1	NannoCCMP1779_4340	[1]
		NoDGTT2	NannoCCMP1779_3705	[1]
		NoDGTT3	NannoCCMP1779_7206	[1]
		NoDGTT4	NannoCCMP1779_9929	[1]
		NoDGTT5	NannoCCMP1779_3915	[1]
		NoDGTT6	NannoCCMP1779_9590	[1]
		NoDGTT7	NannoCCMP1779_3159	[1]
		NoDGTT8	NannoCCMP1779_358	[1]
		NoDGTT9	NannoCCMP1779_10272	[1]
		NoDGTT10	NannoCCMP1779_5368	[1]
		NoDGTT11	NannoCCMP1779_3592	[1]
		NoDGTT12	NannoCCMP1779_3520	[1]
brassicales (plant)	*Arabidopsis thaliana*	*AtDGAT1*	NCBI Ref. Seq.: NP_179535.1	[2]
		AtDGAT2	NCBI Ref. Seq.: NP_566952.1	[2]

mammalia (animal)	*Mus musculus*	*MmDGAT1*	NCBI Ref. Seq.: NP_034176.1	[2]
	Ratus norvegicus	*RnDGAT1*	NCBI Ref. Seq.: NP_445889.1	[3]
	Homo sapiens	*HsDGAT1*	NCBI Ref. Seq.: NP_036211.2	[3]
		HsDGAT2	NCBI Ref. Seq.: NP_835470.1	[3]

Table S5. Information on DGATs sequences used for analysis with PRALINE software.

References:

1. Vieler A, Wu G, Tsai CH, Bullard B, Cornish AJ, Harvey C, Reca IB, Thornburg C, Achawanantakun R, Buehl CJ *et al*: **Genome, functional gene annotation, and nuclear transformation of the heterokont oleaginous alga Nannochloropsis oceanica CCMP1779**. *PLoS genetics* 2012, **8**(11):e1003064.
2. Chen JE, Smith AG: **A look at diacylglycerol acyltransferases (DGATs) in algae**. *Journal of biotechnology* 2012, **162**(1):28-39.
3. Cao H: **Structure-Function Analysis of Diacylglycerol Acyltransferase Sequences from 70 Organisms**. *BMC Research Notes* 2011, **4**:249.
