# Supplementary material for: Nannochloropsis, a rich source of diacylglycerol acyltransferases for engineering of triacylglycerol content in different hosts
Source: Biotechnol Biofuels. 2017 Jan 3;10:8. doi: 10.1186/s13068-016-0686-8 (PMC5210179; doi:10.1186/s13068-016-0686-8)
Supplement: Supplementary file 13 — Additional file 13: Table S1. Primers used for reverse transcription quantitative PCR. [file 13068_2016_686_MOESM13_ESM.pdf]

GENE NAME	GENE ID	$^{(5')}$FORWARD PRIMER$^{(3')}$	$^{(5')}$REVERSE PRIMER$^{(3')}$
NoDGAT1	CCMP1779_3520	GAGCATTTCTGCGTCCGTTC	CCCATCTTGACACAGTCGCT
NoDGTT1	CCMP1779_4340	TTATTTGAAATGTGGTGTATTGC	CTGACAGAAGAGACTATGGT
NoDGTT2	CCMP1779_3705	GCTCACCTCTTCCGTCGTAG	CGGCTCGATTTCGGATGAGA
NoDGTT3	CCMP1779_7206	GTCGTGGCTCTTCCGAGAAA	GAGAGGAAGGATACCGTGCG
NoDGTT4	CCMP1779_9929	GGAGATGTTGGTGGAGAG	GTAGTAGTAGTTGTCGTAGCA
NoDGTT5	CCMP1779_3915	CAGCAAACTGTTCAATGTGG	TAGTAAAGCTCCTCGACCTT
NoDGTT6	CCMP1779_9590	ATGTCCTCCTTCTTGCGTTGGC	GCTATTATTCTTACCGCTGCTACTGC
NoDGTT7	CCMP1779_3159	GGCTGTTCAGTGAGTATCT	CCATTCGTATAAACCCTTTCC
NoDGTT8	CCMP1779_358	CCTCACCATCTGCACCTGAG	TGAAGGGGCTTGGAAGCAAA
NoDGTT9	CCMP1779_10272	CGTTCGTCTTTGGGGAGGAA	CTTTCCGACGAACGCAACTG
NoDGTT10	CCMP1779_3159	CCGGAAATGTGAGACACGGA	CCCACCACCTCGACTGAAAA
NoDGTT11	CCMP1779_5368	CGGAGCCTTTGTTGTTTCGG	TGAGCCACCAACCAGAATCC
NoDGTT12	CCMP1779_3592	ATATCCGTATGTTGTCAAGGT	GAAGCCGTAGGTGAAGAG
NoACTIN	CCMP1779_1821	GCCGTTATTGGATGGATATG	AACAACTCTCCTTCACA
AtACTIN2	At3g18780	TGTGACAATGGTACCGGTATGG	GCCCTGGGAGCATCATCTC

Table S1. Primers used for reverse transcription quantitative PCR.
