# Supplementary material for: Nannochloropsis, a rich source of diacylglycerol acyltransferases for engineering of triacylglycerol content in different hosts
Source: Biotechnol Biofuels. 2017 Jan 3;10:8. doi: 10.1186/s13068-016-0686-8 (PMC5210179; doi:10.1186/s13068-016-0686-8)
Supplement: Supplementary file 14 — Additional file 14: Table S4. Primers used for amplification of sequences of pnoc ox venus vector and pnoc gfp dgtt5pro vector used for transformation of N. oceanica CCMP1779. [file 13068_2016_686_MOESM14_ESM.pdf]

GENE NAME	GENE ID	$^{(5')}$FORWARD PRIMER$^{(3')}$	$^{(5')}$REVERSE PRIMER$^{(3')}$
LDSP 3' UTR	-	GAGCTCGAAAGATCCAAGAGA GACGAG	CTTAAGGTGATGCTGTTGCTCTTTCC
EF PRO	-	CACCTATAGCTACATGGTAGC TAG	TGTTACGAAGTGAGGGTTGAG
VFP	-	GTTAACCAATTGGGGAGCGGC ATGGTGAGCAAGGGCGAG	ACGCGTAGGCCTGCCGCTTCCTTTG TACAACTCATCCATCCCAAGC
GFP	-	CGTAAACGGCCACAAGTTCA	CGCTTTACTTGTACAGCTCGT

Table S4. Primers used for amplification of sequences of *pnoc ox venus* vector and *pnoc gfp dgtt5pro* vector used for transformation of *N. oceanica* CCMP1779; LDSP; lipid droplet surface protein, EF; elongation factor, VFP; venus fluorescent protein, GFP; green fluorescent protein.
