# Supplementary material for: Nannochloropsis, a rich source of diacylglycerol acyltransferases for engineering of triacylglycerol content in different hosts
Source: Biotechnol Biofuels. 2017 Jan 3;10:8. doi: 10.1186/s13068-016-0686-8 (PMC5210179; doi:10.1186/s13068-016-0686-8)
Supplement: Supplementary file 15 — Additional file 15: Table S3. Primers used for amplification of sequences of listed genes without stop codon for cloning into pEarleyGate 101 vector. DGTT5 PRO refers to NoDGTT5 sequence including its native promoter. [file 13068_2016_686_MOESM15_ESM.pdf]

GENE NAME	GENE ID	$^{(5')}$FORWARD PRIMER$^{(3')}$	$^{(5')}$REVERSE PRIMER$^{(3')}$
NoDGTT5	CCMP1779_3915	ATGACGCCGCAAGCCGACATCACCAGCAAGACGA	CTCAATGGACAACGGGCGCGTCTCCCACTCC
NoDGTT5 PRO	CCMP1779_3915	CACCGATAGAAAGTTGATAGGCAA	CCTGAAGATAAAGGAGTTGC

Table S3. Primers used for amplification of sequences of listed genes without stop codon for cloning into pEarleyGate 101 vector. DGTT5 PRO refers to *NoDGTT5* sequence including its native promoter.
